# Distributive Conjugal Transfer in Mycobacteria Generates Progeny with Meiotic-Like Genome-Wide Mosaicism, Allowing Mapping of a Mating Identity Locus

**DOI:** 10.1371/journal.pbio.1001602

**Published:** 2013-07-09

**Authors:** Todd A. Gray, Janet A. Krywy, Jessica Harold, Michael J. Palumbo, Keith M. Derbyshire

**Affiliations:** 1 Division of Genetics, Wadsworth Center, New York State Department of Health, Albany, New York, United States of America; 2 Department of Biomedical Sciences, University at Albany, Albany, New York, United States of America; University of California Davis, United States of America

## Abstract

Horizontal gene transfer (HGT) in bacteria generates variation and drives evolution, and conjugation is considered a major contributor as it can mediate transfer of large segments of DNA between strains and species. We previously described a novel form of chromosomal conjugation in mycobacteria that does not conform to classic *oriT*-based conjugation models, and whose potential evolutionary significance has not been evaluated. Here, we determined the genome sequences of 22 F1-generation transconjugants, providing the first genome-wide view of conjugal HGT in bacteria at the nucleotide level. Remarkably, mycobacterial recipients acquired multiple, large, unlinked segments of donor DNA, far exceeding expectations for any bacterial HGT event. Consequently, conjugal DNA transfer created extensive genome-wide mosaicism within individual transconjugants, which generated large-scale sibling diversity approaching that seen in meiotic recombination. We exploited these attributes to perform genome-wide mapping and introgression analyses to map a locus that determines conjugal mating identity in *M. smegmatis*. Distributive conjugal transfer offers a plausible mechanism for the predicted HGT events that created the genome mosaicism observed among extant *Mycobacterium tuberculosis* and *Mycobacterium canettii* species. Mycobacterial distributive conjugal transfer permits innovative genetic approaches to map phenotypic traits and confers the evolutionary benefits of sexual reproduction in an asexual organism.

## Abstract 2

We find that genome-wide DNA transfer by conjugation in mycobacteria affords bacteria that reproduce by binary fission the same advantages of sexual reproduction, and may explain the genomic evolution of *Mycobacterium tuberculosis*.

## Author Summary

Bacteria reproduce by binary fission, generating two clones of the original; this restricts the genomic diversity of the population, which brings with it inherent evolutionary drawbacks. This problem can be eased by conjugation, which transfers DNA from a donor to a recipient bacterium. Understanding the potential of conjugal DNA transfer for generating genetic diversity is necessary for estimating gene flow through populations and for predicting rates of bacterial evolution. The influence of chromosomal conjugal DNA transfer on mycobacterial diversity has not been previously addressed. Here, we determine and compare the complete genome sequences of independent progeny from bacterial matings between defined donor and recipient strains of *Mycobacterium smegmatis*. We find the resulting hybrid bacteria to be extremely diverse blends of the parental strains, reminiscent of the genetic mixing that occurs through meiotic recombination in sexual organisms. This novel mechanism of conjugation can create genome-wide mosaicism in a single event, generating segments of donor DNA that range from small (∼0.05 kb) to large (∼250 kb), widely distributed around the recipient chromosome. We exploit this mixing by using genetic tools originally developed for finding mammalian disease genes to locate the genes that confer a donor phenotype in *M. smegmatis*. We speculate that similar genomic mosaicism observed in pathogenic mycobacteria arose from conjugation between ancestral progenitor strains.

## Introduction

Sexual reproduction in eukaryotes promotes genetic diversity by increasing gene flow through a population, permitting both the loss of mutant genes and the acquisition of functionally distinct gene alleles. The diversifying potential is further enhanced by crossover events that create new mosaic recombinant meiotic products, which in turn may impart new functionalities not present in either parent. In contrast, bacterial fission provides rapid clonal expansion to fill an environmental niche, but lacks the evolutionary advantages of sexual reproduction. Horizontal gene transfer (HGT) mitigates the diversification constraints of asexual reproduction by mediating limited gene flow through the population. The fundamental forms of HGT include transformation, transduction, and conjugation. Conjugation is considered a major contributor to HGT, as it can transfer more extensive segments of DNA between different species and even kingdoms [1]–[4].

Conjugation describes the unidirectional transfer of DNA from a donor to a recipient, and requires cell–cell contact. Conjugal processes are traditionally plasmid encoded, or encoded by a discrete genetic element integrated into the chromosome. Transfer proteins are generally classified into those that establish and maintain mating-pair formation or those responsible for DNA transfer [5],[6]. These latter proteins recognize and nick the unique origin of transfer (*oriT*) on the plasmid and guide the DNA into the recipient cell. *oriT* is *cis*-acting, and thus, when recombined into the chromosome, it can mediate transfer of chromosomal DNA, as first described for *E. coli* Hfr strains [7]. DNA transfer in *M. smegmatis* displays all of the hallmarks of conjugation: it requires stable and extended contact between a donor and a recipient strain, it is DNase resistant, and the transferred DNA segments are incorporated into the recipient chromosome by homologous recombination [8]. While the process clearly meets the traditional definition of conjugation, the similarities with the classical *E. coli* Hfr system end there [9]–[13]. Mycobacterial conjugation is chromosome—not plasmid—based, and bioinformatic and genetic studies have yet to identify a genetic element that might mediate transfer [14],[15]. In *E. coli*, Hfr transfer always initiates at the sole plasmid-encoded *oriT* site, and the DNA is transferred in a 5′ to 3′ direction, such that only genes proximal and 3′ to *oriT* are inherited at high frequencies [10],[16]. By contrast, in *M. smegmatis*, all regions of the chromosome are transferred with comparable efficiencies as demonstrated by equivalent transfer of a kanamycin-resistance marker regardless of its chromosomal location [11]. This position independence is consistent with the presence of multiple, but ill-defined, initiation sites [17].

Transposon mutagenesis screens provided initial insights into the genetic requirements of transfer [14],[15]. These studies established a prominent role for the Type VII secretion apparatus, ESX-1, in both donor and recipient activity. ESX-1 clearly plays different roles in each cell type. ESX-1 donor mutants are hyperconjugative, suggesting secretion plays a role in negatively regulating transfer activity [15]. By contrast, recipient strain ESX-1 mutants do not receive donor DNA [14]. Although these studies provided novel insights into the functional roles of ESX-1, they did not provide insights on the transfer mechanism, or define what determines the mating type of a cell (either donor or recipient).

Here, as an alternative approach, we examined the products of DNA transfer to better understand this process and its contributions to mycobacterial evolution. We used next-generation sequencing to determine the parental inheritance profiles in transconjugant *M. smegmatis* progeny. The genomic sequence of each of the *M. smegmatis* parental strains has been determined, and the abundant single nucleotide polymorphisms between the two strains indicated that the transferred segments comprising the transconjugant genomes could be mapped with precision. We found that the parental contributions to the transconjugants were much more complex than expected, indicating a surprisingly major role for conjugal DNA transfer in generating genomic diversity. The blending of the parental genomes is reminiscent of that seen in the meiotic products of sexual reproduction. This comparison is validated by our use here of genomic approaches previously developed and applied in sexual reproduction systems to define candidate genes for conjugal mating identity.

## Results

### Transconjugant Genomes Are Highly Mosaic

**Figure 1:**
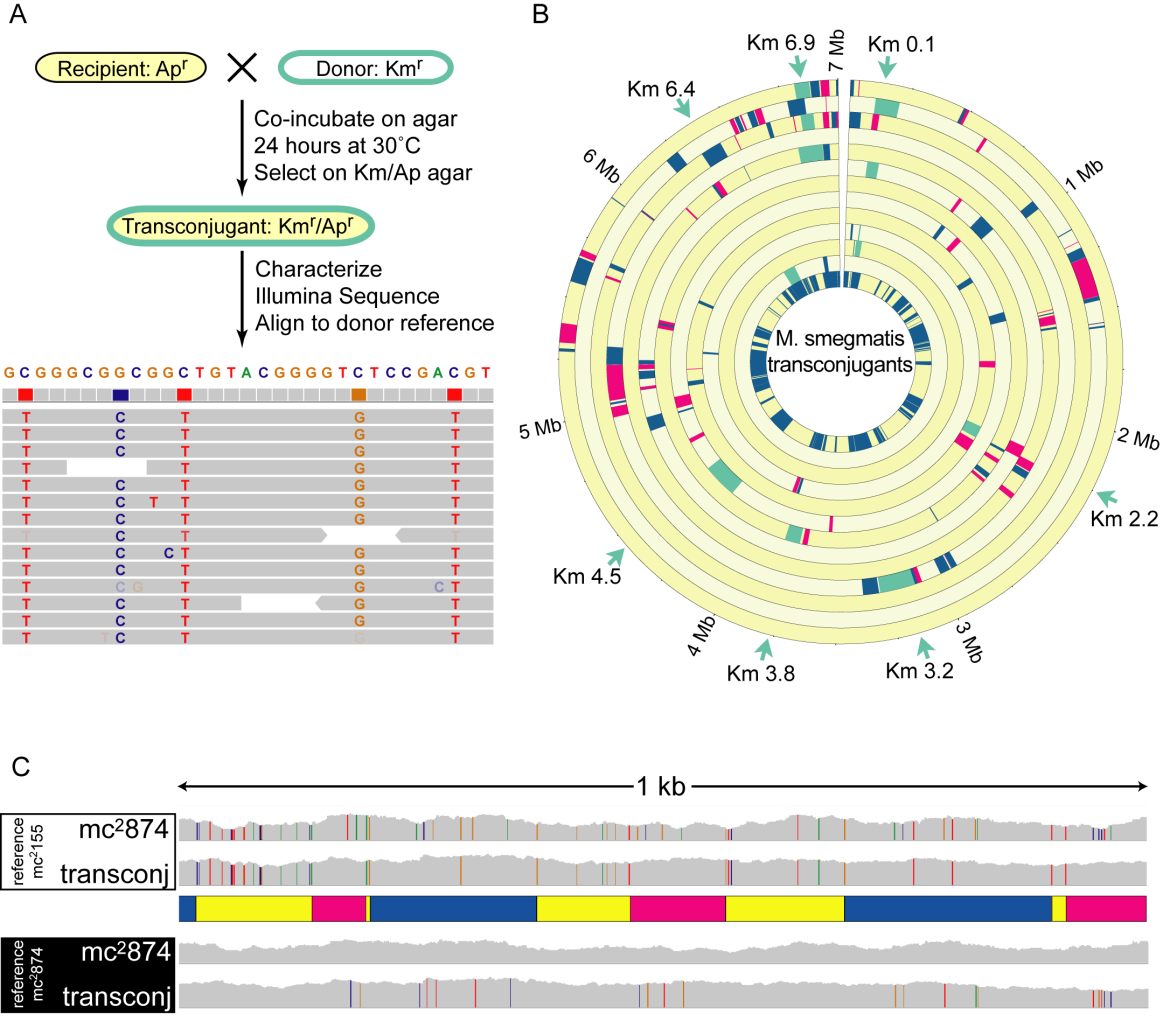
Mycobacterial transconjugant genomes are complex mosaics of their parental strains. (A) Conjugation and genome comparison protocol. Sequence reads for each transconjugant were aligned with the reference donor genome and viewed with IGV. Columns of colored nucleotides mark informative SNPs between the recipient and donor strains, while random colored nucleotides indicate sequence errors. (B) A Circos plot depicts the mosaic nature of 12 *M. smegmatis* transconjugant genomes. mc^2^155 donor DNA segments (alternating blue and magenta, or green) replaced homologous recipient sequences (yellow). Positions of integrated kanamycin genes (*Km*) are shown around the periphery (green arrows), and transferred donor DNA segments containing the *Km* gene are shown in green. Strain nomenclature is based on the genomic location of the *Km* gene in Mb, thus Km0.1 is inserted at coordinate 0.1 Mb in mc^2^155. Strains are from outer to inner circle, respectively: Km6.9e, Km0.1f, Km6.9d, Km3.2, Km6.9c, Km0.1e, Km3.8, Km4.5b, Km2.2a, Km0.1d, Km0.1c, and Km6.4a. The innermost circle is a compilation of all segments of mc^2^155 DNA, showing that almost all regions of the donor chromosome were transferred despite the small sample size. (C) Microcomplexity of parental SNP profiles at some transconjugant recombination sites. Compiled sequence read landscapes are shown for mc^2^874 and one transconjugant (Km6.9e) aligned to the mc^2^155 sequence (top) for a 1 kb segment of the genome (see Table S2, coordinates 470,385–471,385). The presence of informative SNPs (each color represents a different base) indicates recipient sequences, while segments lacking SNPs define donor sequence. Accordingly, parental genotype segments are shown in the schematic below with recipient (yellow) and donor (blue and magenta) genotypes interspersed throughout this 1 kb region. Note that rare cases of isolated recipient SNPs in our designated donor segments are excluded by our stringent criterion requiring two consecutive SNPs to conclusively establish parental origin. The lower panel shows the same sequences aligned to the mc^2^874 sequence, in which the SNPs now indicate donor sequence. This reciprocal alignment confirms the assignment of donor and recipient sequences in the schematic map.

To provide a selectable marker for chromosomal DNA transfer, a kanamycin resistance gene (*Km^r^*) was integrated in the chromosome of mc^2^155, the standard laboratory and conjugal donor strain of *M. smegmatis*. Donor mc^2^155 derivatives that differed in their *Km^r^* insertion site were mated to an apramycin-resistant (Ap^r^) recipient strain, mc^2^874 (Figure 1A). mc^2^874 is an independent isolate of *M. smegmatis* that we have used as a standard recipient strain [8],[18]. Apramycin resistance was episomally encoded to avoid inheritance biases caused by selecting for this gene on the recipient chromosome. From matings between these strains, 12 independent Km^r^Ap^r^ F1 progeny were isolated, and the DNA sequences of their genomes were determined (sequence data deposited in the EBI/ENA database at http://www.ebi.ac.uk/ena/data/view/ERP002619). Our comparative sequence analyses of the parental strains had shown that the circular mc^2^155 and mc^2^874 genomes are collinear, and that they contained abundant single nucleotide polymorphisms (SNPs; averaging one per 56 bp) providing a clear distinction between parental DNA origins (Figures 1A and S1). Individual sequence reads from each transconjugant were aligned with the donor strain genome to identify all transferred donor segments. When evaluating transconjugant sequences, we conservatively required the presence or absence of two consecutive recipient SNPs to define a boundary between recipient and donor sequence tracts, respectively (Figure S2). Donor segments replaced the corresponding recipient sequences, as evidenced by a concomitant localized loss of recipient-specific SNPs in transconjugants. Unique segments of transferred donor DNA, predicted by alignment analyses in transconjugants, were confirmed by conventional PCR and Sanger sequencing (Table S1). Two transconjugants had 11 regions that were merodiploid (approximately equal contributions of donor and recipient SNPs). As this was a resequencing and not a *de novo* sequencing strategy, we cannot determine the precise architecture and location of these regions. These regions did not contain repetitive elements, though it is possible that integration occurred at nonsynonymous sites via microhomology or through mechanisms not requiring homology.

The most striking observation from an alignment of our initial set of 12 transconjugant genomes with the parental genomes was that the transconjugant genomes were broadly mosaic, containing at least two, and as many as 21, separate tracts of cotransferred mc^2^155 DNA embedded in an mc^2^874 background (Figure 1B and Table S2). These separate segments of DNA were acquired in a single cell–cell transfer event, as determined in earlier studies [11]. To our knowledge, this degree of genome-wide diversity is unprecedented in genetic transfer events between bacteria. This contrasts directly with the iconic plasmid-transfer systems in which a single segment of donor DNA linked to *oriT* is inherited [10],[19]. Therefore, we refer to mycobacterial conjugation as distributive conjugal transfer to distinguish it from *oriT*-mediated transfer.

As expected, all transconjugant progeny acquired the selected *Km^r^* gene, along with variable amounts of flanking mc^2^155 DNA (Figure 1B, Km^r^, green segments embedded in yellow recipient DNA). Surprisingly, 5-fold more mc^2^155 DNA was co-inherited in segments that were not selected, and these segments were distributed around the genome with no obvious regional biases (Figure 1B, alternating blue and magenta improve visual discrimination between adjacent tracts; Table S2). The 12 transconjugant genomes analyzed contained from 57 kb to 679 kb (of 6.9 Mb) of mc^2^155-derived sequence. The sizes of the donor segments varied >1,000-fold, ranging from 59 bp to 226 kb (Figure S3 and Table S2), with an average size of 33.8 kb, and a mean of 10 tracts per genome (Table 1).

**Table 1:** Total contributions of donor-derived DNA in transconjugants.

	F1 Recipient (*N* = 12)		F1 Donor (*N = *10)		Backcross (*N* = 6)	
Unit Measured	*N*	%	*N*	%	*N*	%
**Donor**	4,297,500	5.1	8,374,846	12.0	872,006	2.1
**Km^r^**	870,085	20.2	1,093,908	13.1	450,405	51.7
***esx1***	4,399	0.0	1,413,460	16.8	311,489	68.5^a^
**Unselected** b	3,427,415	79.8	6,168,951	73.6	329,884	37.8
**Total segs**	124		166		28^c^	

**Figure 2:**
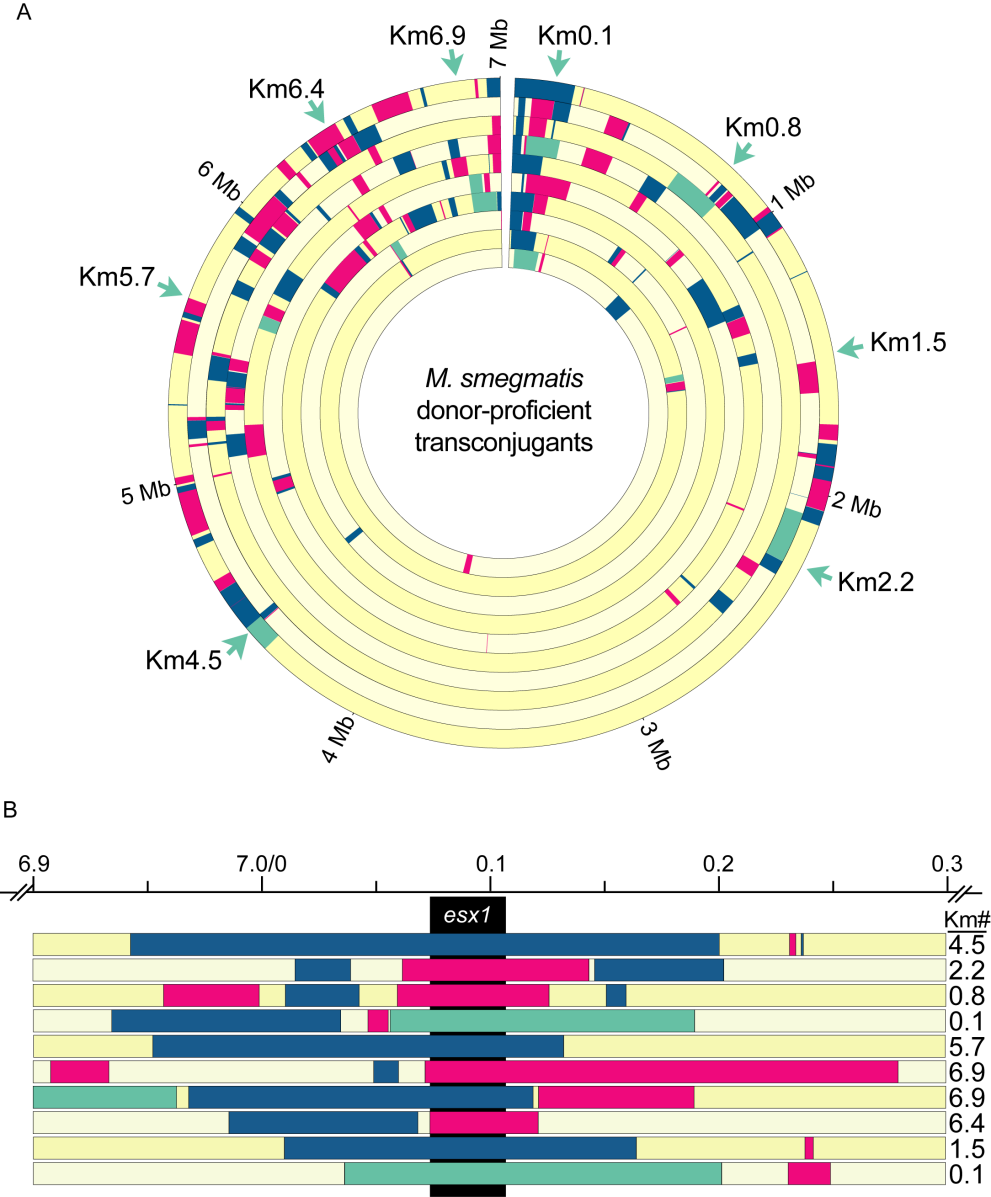
Exploiting DCT to identify *esx1* as a determinant of donor-recipient function. (A) A Circos plot depicts the fragmented genotype of 10 donor-proficient transconjugant genomes. Color key is the same as Figure 1. Strains are from outer to inner circle, respectively: Km4.5a, 2.2b, 0,8, 0.1a, 5.7, 6.9b, 6.9a, 6.4b, 1.5, and 0.1b. (B) An expanded map of the region inherited by all donor-proficient progeny, which includes a single contiguous segment of mc^2^155 DNA encompassing the *esx1* locus (black). Clones are in the same order, outside-to-inside as in (A), and are labeled to indicate the location of the *Km^r^* gene used in selection. Colored bars indicate the extent of DNA inherited from mc^2^155 in the recipient genome (yellow). The *esx1* locus extends from 74,600 to 107,334 bp in mc^2^155.

The total number of base pairs in donor-derived segments was calculated for three transconjugant cohorts (itemized lists appear in Tables S2 and S4). The total DNA can be subdivided into DNA associated with the selected *Km^r^* gene, *esx1*, and unselected DNA. Transconjugant cohorts are recipient-proficient F1 transconjugants (Figure 1); donor-proficient F1 transconjugants (Figure 2), for which *esx1* was enriched by screening for donor function; and backcross transconjugants that are either donor or recipient-proficient (Figures 4 and 5). Donor percentages assume 7 Mb of DNA per transconjugant genome, whereas percentages for segments that spanned *Km^r^*, *esx1*, or were transferred but not selected were calculated per the total amount of donor DNA transferred in that cohort.

a The percentage for *esx1* segments in the backcrosses was calculated for the three donor-proficient derivatives.

b Unselected segments are not contiguous with the donor-derived *Km^r^* gene or *esx1* locus.

c Only three of the transferred segments were unchanged from their ancestral F1 parental boundaries, with the remainder representing subdivided fragments of previously uninterrupted donor tracts.

Some regions showed intricate microcomplexity of multiple inherited segments separated by short intervals of recipient DNA (Figure 1C and highlighted in Table S2). Note that the single-nucleotide discrepancies (colored SNPs) derive from parental inheritance, not *de novo* mutation (see reciprocal parental reference sequence alignments in Figure 1C). These likely resulted from a combination of repair and recombination events occurring between the recipient chromosome and a single molecule of introduced donor DNA, as some segments are separated by only a few base pairs. Regardless of the mechanism, the net effect was to create a localized composite blend of parental contributions at the nucleotide level.

### DCT Facilitates a Genome-Wide Mapping Approach That Identifies a Mating Identity (*Mid*) Locus

The image in Figure 1B shows the extent of mc^2^155 DNA transferred to recipients when selecting for a single event: acquisition of the gene encoding Km^r^. Based on the distributive nature of transfer, we reasoned that we could employ secondary screens of the transconjugants to map any additional genetic trait regardless of its linkage to the *Km^r^* gene. Tracking parental SNPs within a group of individual transconjugants exhibiting a given phenotype should identify those shared SNPs (and parental genes) associated with that phenotype. We have previously observed that a subset of transconjugants become donors, suggesting that these progeny acquired a donor-conferring locus [11]. We hypothesized that an unbiased genome-wide mapping approach would identify a shared segment of mc^2^155 DNA among those progeny encoding this trait. Transconjugants derived from crosses of the differentially marked donor strains were screened for donor ability, and 10 independent donor-proficient transconjugants were identified. We note that mating identity is a mutually exclusive phenotype, and transconjugants exhibit transfer efficiencies comparable to parental strains ([11] and Table S3). Genomic DNA from each donor-proficient transconjugant was prepared and its sequence determined. Comparative sequence analysis showed that all donor-proficient transconjugants, regardless of the location of the *Km^r^* gene in the parent, shared only one segment of mc^2^155 DNA (Figure 2A and Table S4), with the smallest region of overlap encompassing coordinates 74,522 to 119,788 bp (Figure 2B). This result is consistent with transfer of a single 45 kb locus (*mid*) that is sufficient to switch mating identity from recipient to donor in these transconjugants.

This region is not simply a hot spot for integration of acquired DNA, since the 12 recipient-proficient (i.e., did not become donors) transconjugants in Figure 1B were not similarly enriched for this segment of mc^2^155 DNA (compare Figures 1B and 2A, and see below). Closer examination of the region acquired by donor-proficient transconjugants established that they all had inherited a minimal segment of DNA encompassing the mc^2^155 *esx1* locus (Figure 2B, 74,600–107,334 bp, *esx1*_D_, where the subscript differentiates donor or recipient origin). The *esx1* locus encodes a Type VII secretion system [20],[21]. The encoded ESX-1 apparatus assembles in the cell membrane and secretes a specific set of proteins, which, in *M. tuberculosis*, are essential for pathogenicity [22]–[24]. Proteins secreted by ESX-1 lack a signal peptide that would aid in their identification, and the most notable substrate is a heterodimer of two small proteins, EsxB and EsxA. Other proteins encoded within the *esx1* locus and elsewhere in the genome are also secreted through ESX-1, some of which are co-dependent on EsxBA secretion. The functions of most of the proteins encoded by *esx1* genes are unknown, but the overall composition of the *esx1* loci between the parental mc^2^155 and mc^2^874 strains are similar (see below). Although our previous transposon mutagenesis studies have shown that ESX-1 plays an important role in the process of DNA transfer in both donor and recipient strains, mating-type identity is not reversed in ESX-1 mutants [14],[15]. Therefore, the role of ESX-1 in determining mating identity was quite unexpected, and underscores the utility of a “change-of-function” mapping approach.

While all of the donor-proficient transconjugants inherited an intact *esx1*_D_ locus, none of the recipient-proficient F1 strains did. Notably, four of the F1 recipient-proficient strains were derived from the Km0.1 parent, in which only 15 kb separate *esx1*_D_ and the selected *Km^r^* gene. Despite this tight linkage, distributive conjugal transfer readily segregated the *Km^r^* gene and intact *esx1*_D_ locus when appropriately screened, thereby augmenting the mapping resolution (Figure 1B, Table S2, and below). Helpfully, one of these recipient-proficient transconjugants (Km0.1c) inherited parts of *esx1*_D_, excluding these *esx1* genes from *mid* candidacy (*0064–0068* and *0077–0083*, Table S2). These negative correlations affirm the functional dependence of the donor trait on the *mid* genes of *esx1*_D_ and demonstrate the robust nature of distributive conjugal transfer in generating the level of genetic diversity necessary for our mapping analyses.

### Fine Mapping of the *Mid* Locus by a Backcrossing Analysis

**Figure 3:**
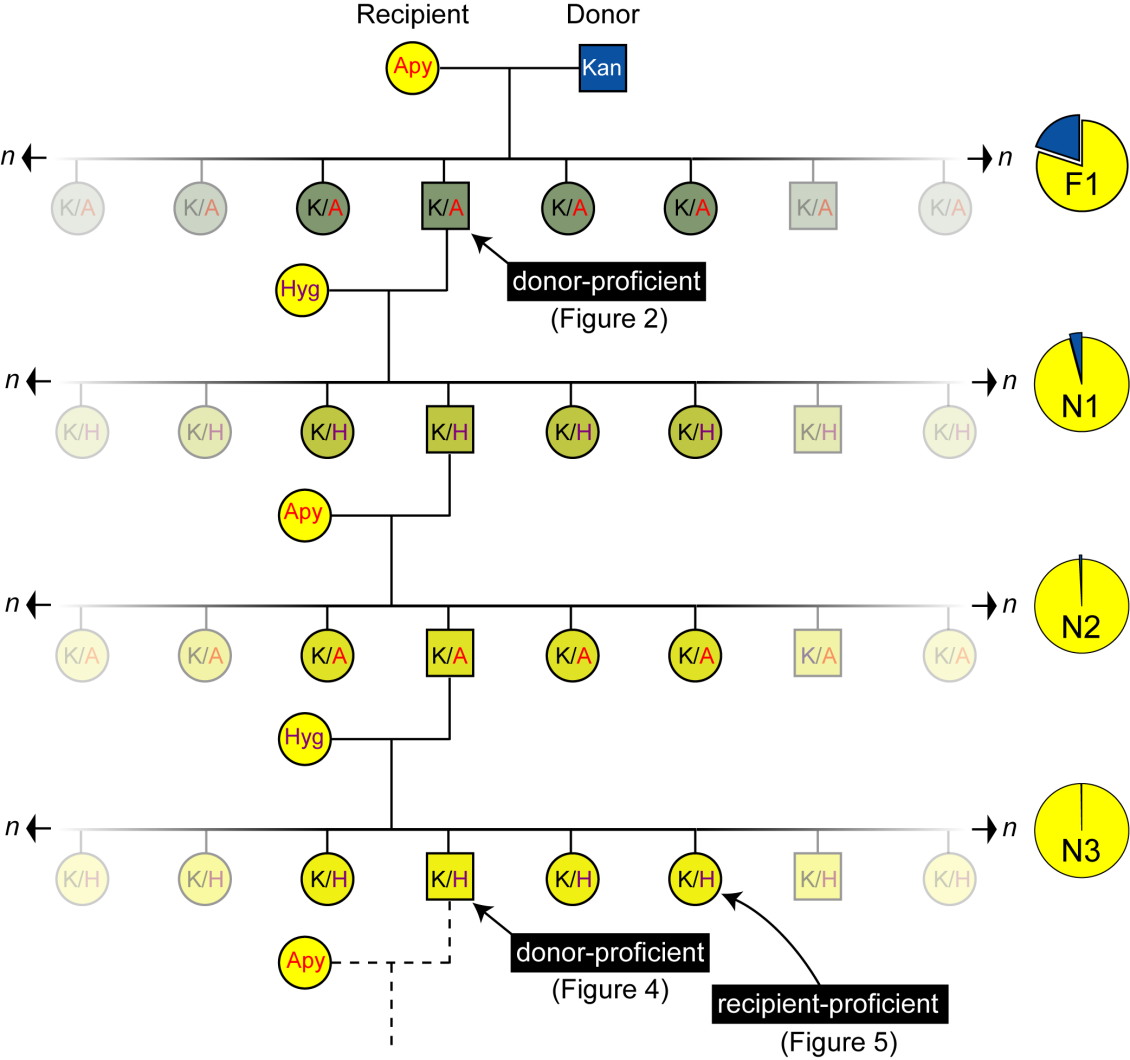
A pedigree showing the backcross introgression strategy. To generate the initial F1 progeny, a kanamycin-resistant (Kan) mc^2^155 donor strain (blue square) was crossed with an apramycin-resistant (Apy) mc^2^874 recipient (yellow circle). The doubly resistant F1 transconjugants (K/A) were screened to identify donor-proficient progeny (green squares; see Figure 2). Donor-proficient F1 derivatives were then backcrossed with a derivative of the original mc^2^874 recipient strain that was marked with a plasmid encoding hygromycin resistance (Hyg). Doubly resistant transconjugants (K/H) were selected to create the N1 generation of transconjugants. As for the F1 stage, the N1 transconjugants were screened to identify donor-proficient progeny (squares) before backcrossing to the apramycin-resistant mc^2^874 recipient to generate the N2 generation. This process was reiterated to genetically purify the donor-determining genes in the mc^2^874 recipient background. Donor-proficient (square) or recipient-proficient (circle) progeny were isolated at either the N3 or the N6 stage, and their genomic DNA was isolated and the sequence determined (see Figures 4 and 5, respectively). The progressive purifying selection of the *Km^r^* and mating identity genes is depicted by the reduced portion of the mc^2^155 DNA (blue sector) through each generation in the mc^2^874 genome (yellow circle) at right, and by the gradual conversion of the progeny background from green to yellow.

**Figure 4:**
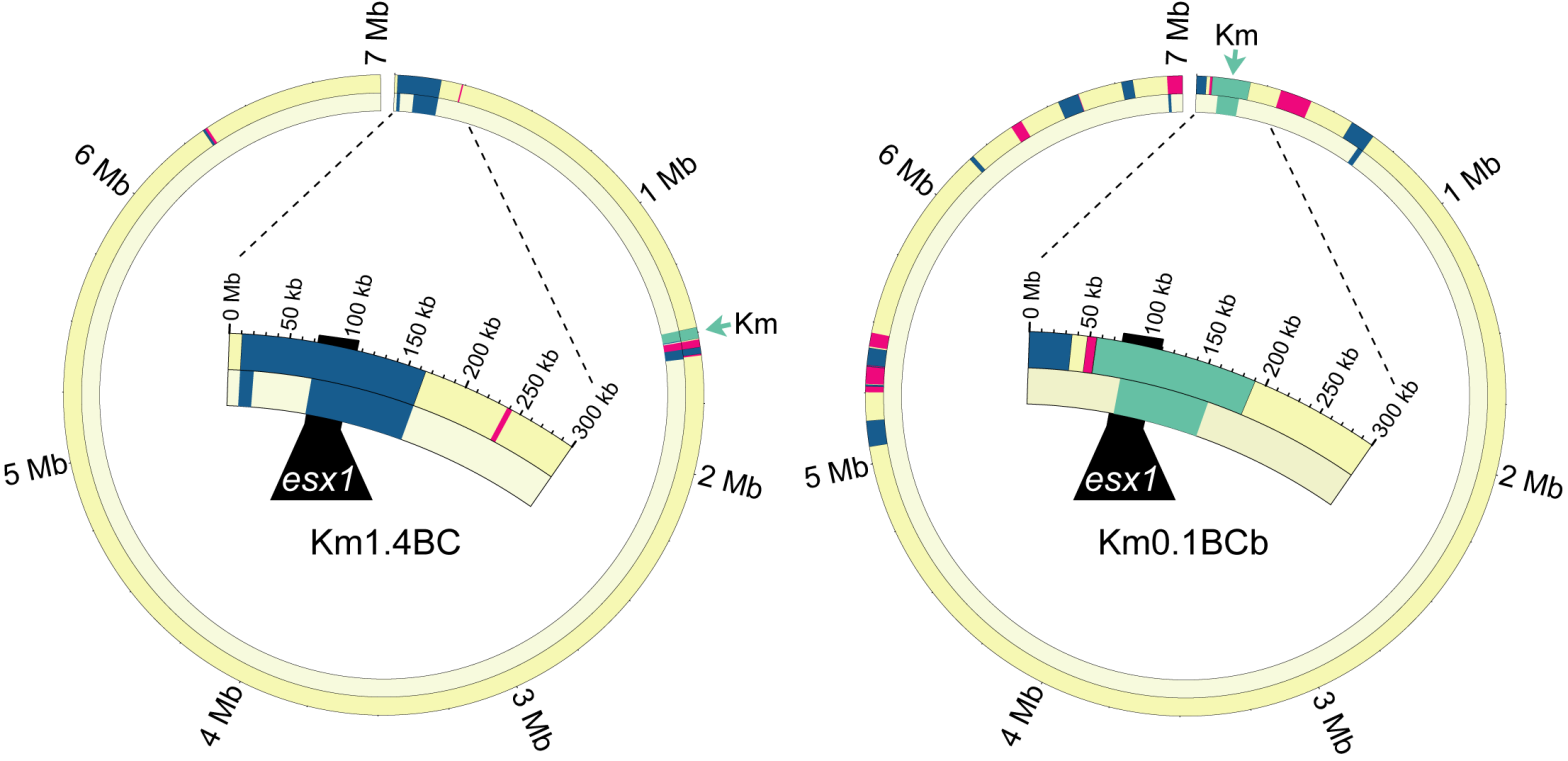
Backcross introgression refines *esx1* as a mating-identity locus in donor-proficient transconjugants. Circos plots of donor-proficient backcross transconjugants showing F1 parental (outer ring) and backcross progeny (inner ring) for each strain pair. Km1.4BC was isolated from N3 progeny and Km0.1BCb from N6 progeny. Backcross (BC) strain names are based on their parent; thus, Km0.1BCb is the second (b), independent transconjugant derived from parent Km0.1. The expanded arc focuses on the *esx1* locus (black). Color key is the same as Figure 1.

In classical genetic studies, fine mapping of a genetic determinant can be achieved by performing successive backcross introgression analyses to genetically purify a locus in a recipient background. We reasoned a similar strategy would achieve two goals: (1) discard mc^2^155 parental genes not required for the donor transfer trait and (2) further narrow the key conjugal *mid* gene region. Six F1 donor recombinants were backcrossed with mc^2^874 recipient derivatives that were marked with a different episomally encoded antibiotic resistance gene (*Hyg^r^* or *Apy^r^*) in successive generations. Introgression entailed co-selection for Km^r^ transfer and the recipient marker to identify transconjugants at each generation (N_x_), and then screening progeny for donor proficiency (Figure 3). Comparative analyses of genomes of three donor-proficient strains showed a purifying selection of the donor-conferring locus and *Km^r^* genes in an otherwise recipient genome (Figure 4, Table S4). In each case, the majority of the F1 mc^2^155 DNA was lost. For example, the F1 parent of Km0.1BCb contained 19 mc^2^155 segments totaling over 869 kb, yet following six backcross generations this DNA was trimmed to three segments totaling 110 kb, most of which encompassed the selected *mid* and *Km^r^* genes (79 kb, Table S4).

**Figure 5:**
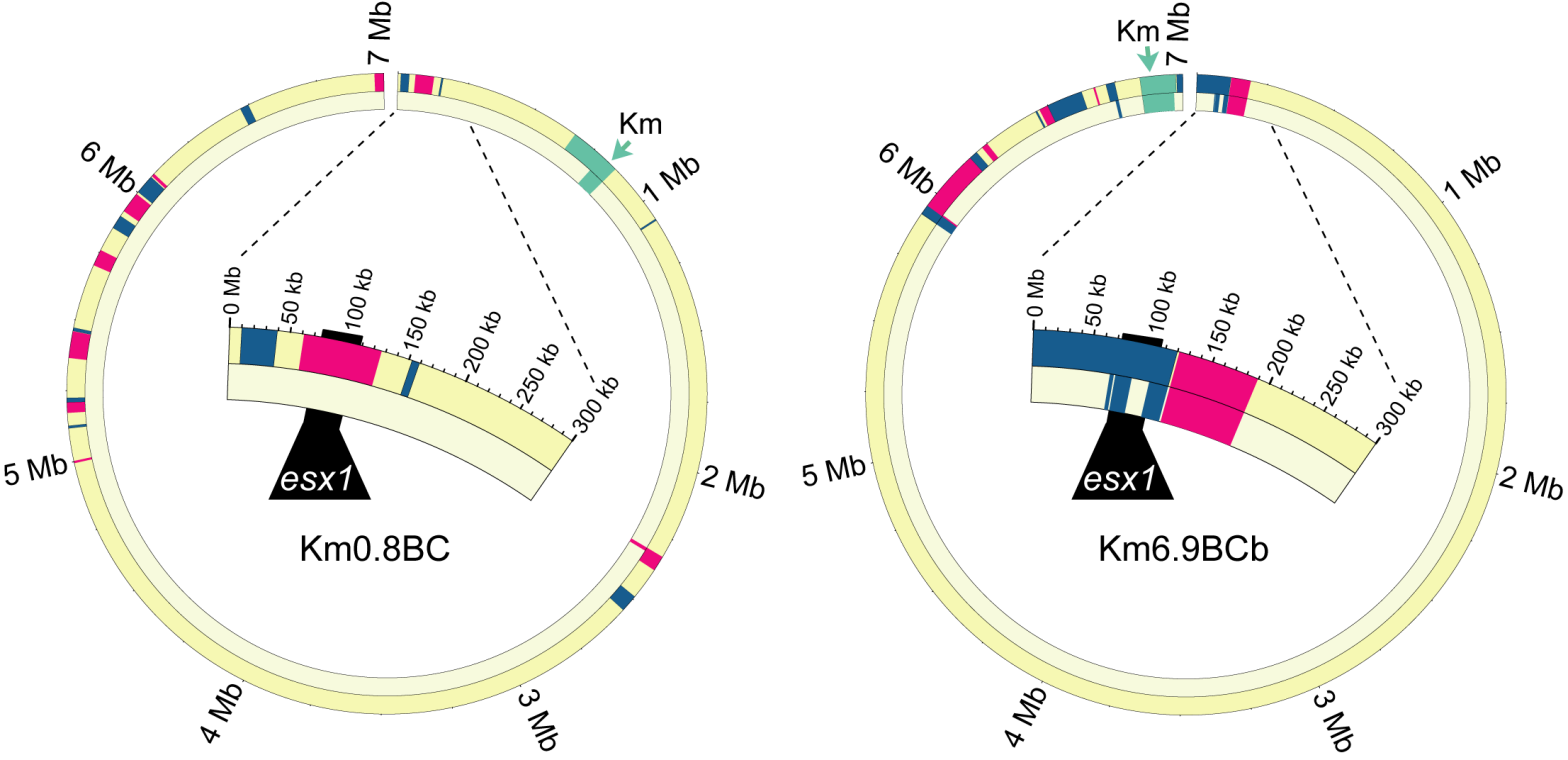
Backcross introgression excludes regions of *esx1* as insufficient for mating identity in recipient-proficient transconjugants. Circos plots of recipient-proficient backcross transconjugants showing F1 parental (outer ring) and backcross progeny (inner ring) for each strain pair. In the third backcross step, none (Km0.8BC) or part (Km6.9BC) of *esx1* was transferred to the isolates shown, coincident with reversion to a recipient phenotype. The part of mc^2^155 *esx1* present in Km 6.9BC indicates that these mc^2^155 genes are insufficient to confer donor identity. Nomenclature and color codes are the same as in Figure 4.

**Figure 6:**
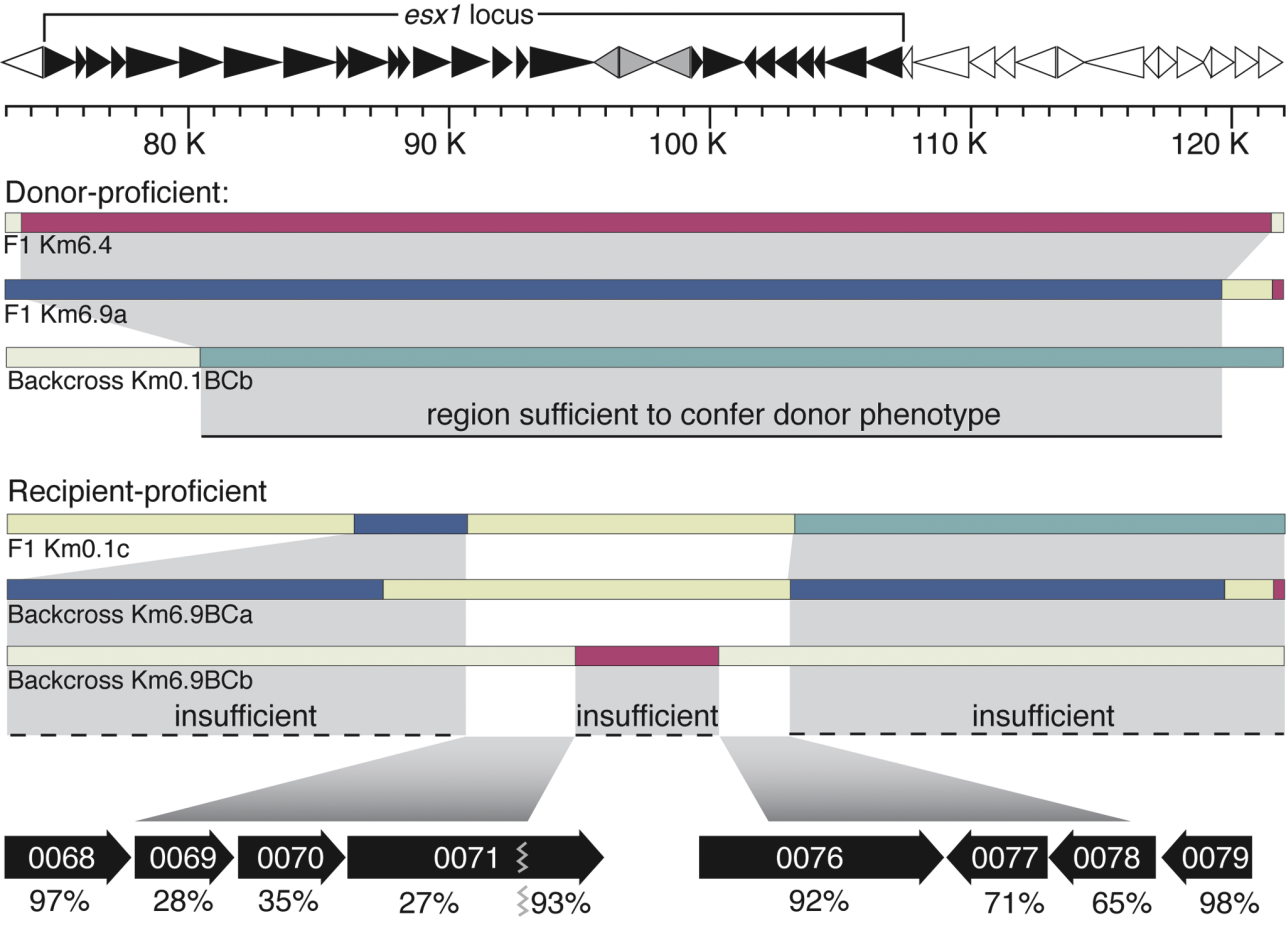
The *mid* locus within *esx1*, as defined by the F1 association mapping and backcross introgression analyses. A schematic guide encompassing 73 kb to 122 kb of the mc^2^155 reference genome, including the *esx1*_D_ locus genes (black filled, *ms0055* at 74.6 kb through *ms0083* at 107.3 kb). A repetitive IS element cluster absent in the recipient (*ms0072–0074*) is gray-filled. Below are key clones from crosses that progressively defined the *mid* gene candidates. Donor-proficient transconjugant clones had inherited mc^2^155 sequences sufficient to convey the donor phenotype. Recipient-proficient transconjugants inherited mc^2^155 sequences that were insufficient to impart the donor phenotype. Considered together, the key *mid* candidate regions span 6,923 bp of mc^2^155 DNA, from 90,697 to 94,949 and from 100,295 to 102,966. These regions span *esx1_ms_* genes *ms0069–0071* and *ms0076–0078* as shown in the expansion at the bottom. The amino acid identities between the encoded proteins of mc^2^155 and mc^2^874 are notably low for the left region, consistent with functional disparity.

As expected, backcross matings also resulted in recipient-proficient progeny, several of which were also sequenced (Figure 3). Coincident with a reversal of mating identity, the *esx1*_D_ locus failed to transfer. One recipient strain, Km0.8BC, retained only 75 kb of mc^2^155 DNA of the 920 kb originally present in the F1 parent (Figure 5, Table S4). Analyses of two recipient-proficient strains derived from independent F1 Km6.9 parents further refined the region of interest. Km6.9BCa included donor genes *0055D–0067D* and *0079D–0083D* and Km6.9BCb contained genes *0072–0075D* (Figures 5 and 6, Table S4). Thus, these *esx1*_D_ genes are insufficient to confer a donor phenotype. Taken together, the mapping data identify *esx1* genes in *0068D–0071D* and/or *0076D–0078D* as being critical for determining mating identity. Ongoing studies requiring multiple, precise, targeted gene swaps will identify the key gene(s).

While most *esx1* gene products are highly conserved among mycobacterial species, *M. smegmatis* proteins 0069, 0070, and the N-terminal two-thirds of 0071 have notably low amino acid identity between donor and recipient orthologs (Figure 6 and Figure S4) [14] and are therefore good candidates for switching mating identity. The proteins encoded from this region are not predicted to contain an obvious motif or domain that would provide mechanistic insight into their role in conjugation. However, the location of the *mid* genes within *esx1* suggests that the encoded proteins modify ESX-1 structure or function, to perhaps affect cell–cell communication or physically mediate DNA transfer.

## Discussion

We used next-generation sequencing to examine transconjugant genomes and found that mycobacterial conjugation generates highly mosaic genomes created by a robust distributive conjugal transfer process. Transconjugants acquired large amounts of donor DNA (some exceeding one-fourth of the transconjugant genome; Table S4, Km4.5a), in varied segment sizes (spanning four orders of magnitude) that were distributed around the genome. We exploited these characteristics of distributive conjugal transfer (DCT) to map mating identity genes of *M. smegmatis*.

**Figure 7:**
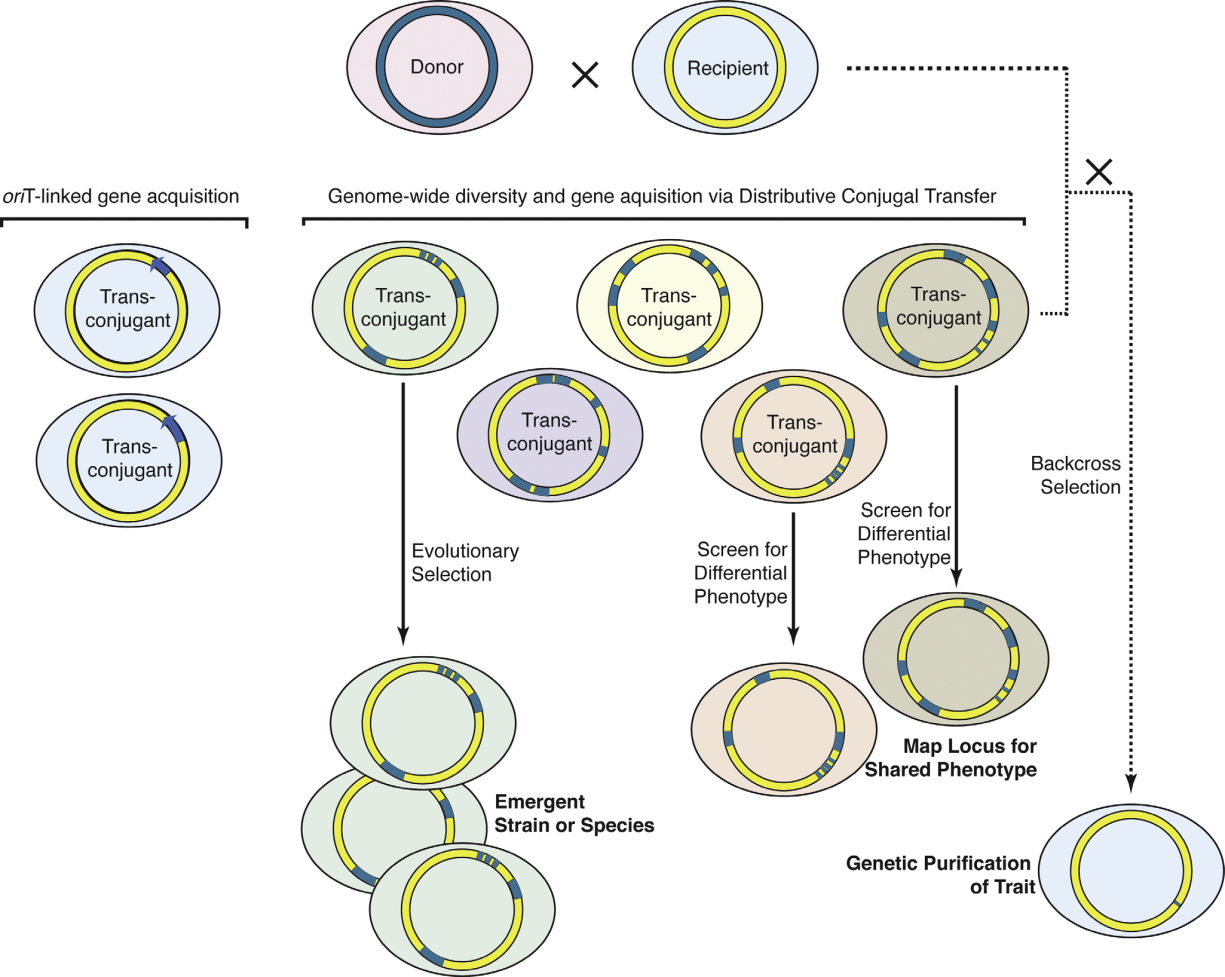
Graphic summary of the evolutionary and gene mapping potential of distributive conjugal transfer in comparison to *oriT*-mediated transfer. The parental donor and recipient strains are schematically shown at the top, with their native chromosomes (blue and yellow circles, respectively) that confer different phenotypes (pink and blue backgrounds, respectively). Co-incubation of the donor and recipient strains on solid media (agar plates) or in a biofilm, permits conjugation. For *oriT*-mediated transfer (left), all transferred segments of DNA are linked to *oriT*, which limits the extent of genetic diversity among the transconjugants. This contrasts with distributive conjugal transfer (DCT), wherein random segments of the donor chromosome are transferred to the recipient, generating unique transconjugants. Each transconjugant has a novel genotype that confers a unique phenotypic profile (different colored background). Importantly, multiple rounds of *oriT*-mediated transfer events with different donors would be required to approach the variation observed from a single DCT event. Under certain conditions, any transconjugant phenotype may have a growth advantage over other transconjugants and the parental strains. Such evolutionary selection can give rise to emergent strains or species. Transconjugants that share a specific phenotypic trait can be sequenced to identify SNPs that mark a shared genomic region associated with that trait. An F1 transconjugant with a specific donor-derived trait can be repetitively backcrossed with the recipient strain to introgress the functional donor gene into the recipient background.

Hfr transfer in *E. coli* is initiated from the unique *oriT* and results in transfer of a single segment of the donor chromosome [9],[19],[25]. Thus, while the recipient acquires new genetic information, that new information is limited to DNA immediately adjacent and 3′ to *oriT* (Figure 7, left). Genetic analyses and an understanding of the RecBCD recombination machinery suggest that a single segment is integrated into the recipient chromosome via a recombination event occurring at each end of the transferred DNA molecule [16]. To our knowledge, whole genome sequencing has not been reported for Hfr– transconjugants, preventing a detailed comparison of the two conjugation systems. Thus, our study provides the first genome-wide analysis of bacterial conjugal transfer. In contrast to *oriT*-mediated transfer, the complex inheritance profiles exhibited by mycobacterial transconjugants suggest stochastic co-transfer from multiple origins, as previously predicted [17]. Based on our genome sequence data, we speculate that random chromosomal DNA fragments are generated in the donor, some of which are co-transferred into the recipient strain where they replace recipient sequences through homologous recombination. An alternative scenario is that a single large DNA molecule is transferred, which is processed into smaller segments before their integration into the recipient chromosome by homologous recombination. This scenario seems less likely as we would have expected to identify some transconjugant progeny containing exceedingly large chunks of donor DNA (3–4 Mb) integrated into the chromosome. These would have resulted from recombination close to the ends of the transferred molecule, before creation of small segments. This latter scenario is also less consistent with our previous observations, which indicated that the donor chromosome contained multiple initiation sites and that the efficiency of gene transfer was location-independent. We have considered examining boundary sequences to determine whether they provide insight on the mechanism of conjugation. However, there are multiple factors influencing boundary regions, which together prevent a unifying mechanistic insight. For example, the actual breakpoints generated by conjugation are almost certainly lost as the boundaries are driven by the requirement for homology and by different recombination mechanisms mediating integration, as evidenced by inheritance of both regions of microheterogeneity and single large integration events.

Mycobacteria encode multiple nonredundant recombination pathways (RecBCD, AdnAB, and nonhomologous end-joining), but are not known to encode a mismatch repair system [26],[27]. We postulate that homologous recombination mediated by AdnAB is likely responsible for the simple crossover events, which is consistent with the absolute requirement for RecA in DCT [17]. However, this form of homologous recombination alone seems insufficient to explain regions of microcomplexity. The clustered proximity of recombinant tracks indicates that an imported donor segment initially encompassed the entire region, but the mechanism underlying the internal mosaicism is unclear. Characterization of the mechanism and the enzymes behind this process will require careful directed approaches using defined recombination mutants.

Every facet of the transfer process contributes to the genetic complexity of the transconjugants (Figure 7). The large number and distributive character of the transferred segments, combined with the microcomplexity in some tracts, makes each transconjugant uniquely different from the others, as well as from the parental strains. The widely varied sizes of the transferred segments allows transconjugants to acquire both major changes, potentially bringing in entire operons encoding biological pathways, and minor nucleotide substitutions that provide subtle diversity, which could, for example, modify the activity or interaction specificity of an enzyme. Multiple pan-genomic changes that typically accompany evolution of bacteria are assumed to be a serial accrual of HGT and spontaneous mutation events (Figure 7). By contrast, a single step DCT event between two single cells generates a transconjugant strain that is a mosaic blend of the parental genomes, and not merely an incrementally altered derivative. Thus, distributive conjugal transfer provides an unparalleled mechanism for quickly generating tremendous genetic diversity, which rivals that seen in sexual reproduction [28].

Recent genome-wide studies of naturally competent strains provide an interesting contrast between the progeny of transformation and conjugation [29]–[32]. In these studies, nonselected segments of DNA were also observed around the recipient chromosome and thus contribute to variation. Microcomplexity in these segments suggested that, as for DCT, integration of transformed DNA was mediated by both recombination and/or repair machinery. However, the nonselected segments were significantly smaller (1–4 kb, depending on the species) than those described here, which average 49 kb and can be as large as 249 kb (Table S4, Km4.5b: 6,942,375–202,798). The limitation on recombination sizes in pneumococci correlated with an underrepresentation of large insertions, which together argued that transformation led to genome reduction and was unlikely to act as a mechanism for uptake of accessory loci [29]. The large DNA segments acquired via DCT, in contrast, facilitates inheritance of novel operons and genes. For example, one large recombination tract introduced a contiguous stretch of ∼55 kb of nonhomologous donor-derived DNA into the transconjugant chromosome (Km6.9b). Perhaps an example more functionally pertinent to our work was an insertion–deletion exchange observed in the divergent *mid* candidate region of *esx1* in transconjugants switched to donors (Figure S5).

We have demonstrated conjugal DNA transfer in additional naturally derived *M. smegmatis* strains [8], indicating a broader presence for mycobacterial distributive conjugal transfer. The rough-colony morphology members of the *Mycobacterium tuberculosis* complex (MTBC) exhibit extremely low genetic variation, suggesting that they do not undergo HGT, are evolutionary young, and resulted from a recent clonal expansion [33]. However, there is now convincing evidence for HGT among *M. canettii*, and other smooth-colony MTBC strains, which display genome-wide mosaicism, although the precise mechanism(s) of HGT are unknown [34],[35]. Based on sequence comparisons, it was proposed that *M. canettii* strains are extant members of a genetically diverse MTBC progenitor species, *M. prototuberculosis*, whose members underwent frequent HGT [34],[36],[37]. The unspecified HGT process underlying that mosaicism is presumed to result from a series of sequential transfer events. However, based on our studies, distributive conjugal transfer involving the ancestral *M. prototuberculosis* offers a plausible and parsimonious explanation for the remarkably similar mosaicism observed among the extant *M. canettii*. We could envision that distributive conjugal transfer in *M. prototuberculosis* rapidly incorporated the necessary blend of parental genotypes that drove the emergence of the pathogenic, rough-colony morphology species, like *M. tuberculosis*, allowing their subsequent clonal expansion. Moreover, if DCT drove these postulated HGT events, the evolutionary clock for *M. tuberculosis* is likely much shorter because of the capacity of DCT to generate genome-wide mosaicism in a single step. Given the widespread nature of conjugation, we speculate that distributive conjugal transfer also occurs in other bacteria, conferring similar evolutionary benefits.

The characteristics of mycobacterial distributive conjugation suggested to us that tools developed for mammalian genetics could be applied here. Using a eukaryotic-style genome-wide association mapping approach, we mapped the mating identity locus (*mid*) for mycobacterial conjugation (Figure 7). Similarly, we applied a backcross introgression strategy to refine the mapping and to purge extraneous mc^2^155 sequence (Figure 7). The purifying selection of successive backcross generations effectively introgressed the mc^2^155 *mid* locus into the mc^2^874 background; this created a strain that was nearly isogenic to the mc^2^874 parent strain, but which now functioned as a conjugal donor. We note that the hybrid *esx1* loci produced by distributive conjugal transfer have not been disabled (as in transposon mutagenesis screens), and still encode functional ESX-1 secretory apparatuses that secrete the major ESX-1 substrates (Figure S6). The un-annotated theoretical proteins encoded by the *mid* candidate genes bear no overt resemblance to those known to be involved in conjugation in other bacteria. Their association with the *esx1* locus suggests that Mid proteins modify the ESX-1 secretion system, are secreted by ESX-1, or interact with other ESX-1–secreted substrates. The next step in their functional assessment will likely result from an extension of this work to identify which protein(s) or protein motifs are necessary and sufficient to impart conjugal sex identity. Interestingly, orthologs for the *mid* candidate genes are found in the sequenced genomes of other environmental mycobacteria, suggesting a possible ongoing role for distributive conjugal transfer in gene flow between mycobacteria. Orthologs of these *mid* candidates are not apparent in the *esx1* locus of *M. tuberculosis*, consistent with our speculative model that the MTBC represents a clonally expanded product of distributive conjugal transfer, not necessarily an active participant in this process. Nevertheless, recent evidence from genome sequencing comparisons indicates that some form of genetic exchange has occurred between *M. tuberculosis* and *M. canettii* [35].

While we applied DCT to map *mid* genes, in principle any genetic trait that differs between the parental strains can be mapped using this genome-wide mapping strategy. For example, mc^2^155 and mc^2^874 grossly differ in colony morphology, biofilm formation, and phage susceptibility, any of which could have been scored as a change of function in the recipient and mapped by DCT. Similarly, biochemical differences between these strains could be discerned through simple, high-throughput assays. We recognize that more traditional approaches for mutagenic loss-of-function mapping [38],[39] will remain important in mycobacterial studies, but this new application of conjugation now allows any phenotype that differs between a mating pair to be unambiguously mapped.

Our analysis of distributive conjugal transfer (DCT) in *M. smegmatis* has practical and conceptual ramifications. It brings new tools to mycobacteriology, including those traditionally used exclusively in eukaryotic genetics. It also shows how bacterial evolutionary time scales can be compressed by generating incredible genetic diversity in a single step. Identifying the necessary components, such as *esx1* and *mid*, will help to elucidate the mechanism, to allow modification of the system, and to computationally identify bacteria that actively participate in DCT—or engineer them to do so. Our previous finding of DCT in a mixed biofilm [40] underscores the importance of predicting how prevalent DCT may be in nature, for a more accurate interpretation of metagenomic datasets and to model gene flow through bacterial populations. Regardless of these secondary ramifications, our primary finding of the tremendous genomic variation generated by DCT takes a significant step toward bringing the evolutionary benefits of sexual reproduction to bacteria.

## Materials and Methods

### Mycobacterial Strains and Conjugation

*M. smegmatis* donor strains were derivatives of the laboratory strain, mc^2^155 [41]. Each derivative has a *Km* ^R^ gene inserted at a unique location in the chromosome [11], which was mapped by DNA sequencing the flanking DNA and alignment to the mc^2^155 genome sequence (http://cmr.jcvi.org/tigr-scripts/CMR/GenomePage.cgi?org=gms), or the draft genome of the recipient (GenBank CM001762). The recipient strain mc^2^874 [18],[42] was transformed with a plasmid encoding either apramycin or hygromycin resistance to allow counterselection against the donor. *M. smegmatis* strains were cultured at 37°C in Trypticase Soy Broth with 0.05% Tween80, or on Trypticase Soy Agar (TSA) plates. Antibiotics were added at 100 µg/ml (apramycin), 100 µg/ml (hygromycin), and 10 µg/ml (kanamycin). DNA transfer experiments were carried out as described previously selecting for dual-resistant transconjugants [8]. To allow selection in the reiterative backcrosses, the recipient strain was alternated between that encoding either apramycin or hygromycin resistance. Each independent transconjugant was assayed in subsequent mating experiments to determine whether they were donor or recipient, in parallel with positive controls. As we have observed previously [8], this phenotype was mutually exclusive. Donor transfer frequencies were determined based on the average of three, independent mating experiments as described previously [8]. Zero transconjugants were obtained with recipient strains, below the sensitivity threshold of one event per 10^8^ cells [8].

### Genomic Sequencing and Analysis

Transconjugants were colony purified, and genomic DNA was prepared and then subjected to whole-genome DNA sequence analysis at the Institute for Genome Sciences (IGS), U. Maryland, using paired-end Illumina technology. The sequence coverage for each genome was between 50-fold for F1 progeny and ∼1,000-fold for backcross strains. Sequence reads were mapped to the mc^2^155 reference sequence by IGS. Single nucleotide polymorphisms (SNPs) or sequence gaps were identified using the Integrative Genomics Viewer (IGV) sequence viewer [43] to define genomic regions of different parental origins. Boundaries of recipient- and donor-derived segments were recorded as the last recipient SNP observed with a minimum of two consecutive SNPs defining parental identity (Figure S2). A donor segment unique to each transconjugant was identified to confirm accuracy of the aligned sequence reads. Primers were designed to specifically amplify these segments, and the amplified products were cloned and sequenced (Table S1) to confirm that donor SNPs had been inherited by the recipient. A compilation of the donor and recipient segments from each transconjugant was projected onto the circular mycobacterial donor chromosome reference sequence, arranged as concentric circles of a Circos plot [44], with color optimization guided by ColorBrewer (Cynthia Brewer, The Pennsylvania State University). Collinearity of the donor and recipient genome was determined using Mauve, a program that was also used to identify SNPs and in/dels [45],[46]. All sequence data have been deposited at the European Nucleotide Archive at http://www.ebi.ac.uk/ena/data/view/ERP002619.

## Supporting Information

Figure S1 Genome collinearity of the parental strains, mc^2^155 (donor) and mc^2^874 (recipient). The circular genomes of the parental strains are depicted in linear form and aligned. Genome sequences for mc^2^874 were obtained by combining reads from one Illumina and two 454 paired-end libraries (GenBank CM001762). Sequence data are deposited in the EBI/ENA database at http://www.ebi.ac.uk/ena/data/view/ERP002619. A *de novo* build was assembled into a scaffold, and this nucleotide sequence was aligned (Mauve 2.3.1) with the GenBank/JCVI sequence for mc^2^155 (CP000480.1) [45],[46]. This figure shows the alignment viewed at Mauve's default, highest stringency setting, thereby displaying even small interruptions. Locally Collinear Blocks (LCBs) are independently colored, with the largest five LCBs comprising nearly all of each genome, and maintaining their order and orientation. The crossed lines between each map indicate modest rearrangements. The sequence data identified 122,186 SNPs (∼1 every 56 bp) between the donor and recipient sequences allowing for easy discrimination between donor and recipient DNA in the transconjugants (see also Figures 1A and S2).
(TIF)

Figure S2 IGV image illustrating donor/recipient junction assignment in a transconjugant. Sequence reads from the recipient strain mc^2^874 and a transconjugant are shown aligned to the mc^2^155 reference sequence. A gray bar indicates an individual Illumina sequence read, with the arrow indicating the direction of each read. For simplicity, a depth of 10 reads is shown here, but the average read depth was approximately 50- to 1,000-fold. Gray sequence indicates identity between the sequenced clone and the mc^2^155 reference genome. Single nucleotide polymorphisms (SNPs) present in sequenced strains appear as colored bases in each read that align vertically with the corresponding polymorphic mc^2^155 nucleotide. The total SNP content in this 649 bp region is revealed upon mc^2^874 recipient alignment with the mc^2^155 reference. Recipient sequence in the transconjugant is conservatively defined by the presence of two consecutive SNPs, and is indicated by the yellow bars below. The left boundary of the replacement donor sequence tract lies between the last recipient SNP present (green) and the next missing SNP (red); as intervening regions match the reference sequence (gray coloration), the donor segment is designated to extend from SNP to SNP, as indicated by the blue bar below. Note that to more clearly discern closely localized donor tracts on the lower resolution Circos plots, successive donor tracts were alternately colored blue or magenta.
(TIF)

Figure S3 Distribution of donor tract sizes identified in transconjugant genomes. The calculated donor-derived tract sizes for the initial 12 transconjugants are graphically displayed (blue bar represents donor segment length).
(TIF)

Figure S4 Pairwise alignments of Mid candidate protein sequences between donor and recipient strains of *M. smegmatis*. A conceptual translation of the six open reading frames comprising the *mid* candidate regions defined by the combined mapping approaches were globally aligned using a Needleman-Wunsch algorithm (http://www.ebi.ac.uk/Tools/services/web_emboss_needle/). Immediately below each protein identifier (bold text) are input parameters and output statistics. In each alignment, the upper sequence represents the mc^2^155 (donor) sequence and the bottom is the mc^2^874 (recipient) sequence. The degree of amino acid conservation is indicated between paired residues: vertical line (identical), dots (similar), or nothing (dissimilar). Horizontal lines represent gaps created by the algorithm to maintain alignment. Similarity statistics for the divergent N-terminus and conserved C-terminus of Msmeg_0071 are listed separately following the full-length alignment of this protein.
(DOCX)

Figure S5 In/dels are transferred in DCT. Whole genome alignment analysis of the mc^2^155 and mc^2^874 parental strains by Mauve identified 694 in/dels of >18 bp. The 3′ end of the *esx1* locus was identified by Mauve as having insertions in mc^2^155 (i.e., deleted or divergent in mc^2^874, indicated by red bars above). Sequence reads aligned to the donor reference viewed in IGV verified that no sequence reads from mc^2^874 (top alignment, yellow background) mapped to the mc^2^155 reference sequence in this region, consistent with in/del status. This ∼9 kb region includes donor genes *Ms0069* through *Ms0071* and a cluster of defective IS elements (*Ms0072–0075*), displayed at the bottom of the IGV window. Donor sequences from a donor-proficient transconjugant (middle alignment, blue background) have replaced this recipient in/del region, showing that novel sequences can be acquired and incorporated by DCT. Note that reads spanning IS elements in this transconjugant have a lower mapping score (light-shaded reads) because they could map to multiple sites in the genome. Recombination events can occur close to in/del regions, as illustrated by the donor reads in *Ms0068* derived from the recipient-proficient transconjugant at the bottom.
(TIF)

Figure S6 A Western analysis shows that hybrid transconjugants, like their parents, still secrete EsxAB. Culture filtrates and cell pellets were collected as described previously [47]. Following concentration, equivalent cell volumes of each sample were loaded and separated on a 4–12% gradient SDS-PAGE gel. Proteins were transferred to a PVDF membrane and then probed with polyclonal antibodies to detect EsxB. EsxB is secreted by the wild-type strain MKD8 and is therefore detected in both the supernatant (2) and cell pellet (3). In a strain containing a transposon insertion in *Ms0062*, EsxB is not secreted (4) and is found exclusively in the pellet (5). In transconjugant Km0.1c (Table S2), which contains a mosaic *esx1* region, EsxB is found in the supernatant (6) and the pellet (7) as for wild-type. Protein standards are shown in lane 1 and are listed in kilodaltons.
(TIF)

Table S1 Primers used to verify transferred donor SNPs in transconjugant genomes. The primers, their genome coordinates, used for each transconjugant are listed. Sanger sequencing of the PCR product verified the presence of uniquely transferred donor SNPs in transconjugant genomes. Multiple informative SNPs present in each amplicon to facilitated unambiguous parental origin identification. Two PCR clones were sequenced from each transconjugant strain to avoid potential complications from PCR errors.
(DOCX)

Table S2 Donor and recipient boundary addresses as mapped to the reference sequence. Transconjugant clone name appears along the left margin. Strain nomenclature is based on the genomic location of the Km gene insertion (in Mb); thus, Km0.1 represents the mc^2^155 derivative with an insertion at coordinate 114 kb. A lower case subscript indicates transconjugants derived from independent crosses using the same parental donor. Donor segments are mapped as where the last consecutive recipient SNP is present (donor begin) to where the next consecutive recipient SNP is detected (donor end). The length of the intervening donor tract (donor size), the total amount of donor DNA in each transconjugant (total donor), and the length of recipient DNA separating adjacent donor tracts are shown (separation). The segments containing the selected kanamycin resistance gene are highlighted in green. Totals for the cohort appear at the bottom. Note that donor regions separated by less than 1 kb are boxed in blue highlights to indicate they may be due to multiple, internal recombination events of a larger transferred segment, or a single recombination modified by mismatch repair.
(XLSX)

Table S3 Transfer frequencies of F1 transconjugants. Transfer frequencies are the number of transconjugants divided by the number of donor cells. These frequencies are the average of at least two independent matings, which were carried out in parallel with a positive control (MKD6 and MKD8, [8]). The threshold of detection is ∼1 transfer event per 10^8^ donor or recipient parental cells.
(DOCX)

Table S4 Donor and recipient boundary addresses as mapped to the reference sequence for donor-proficient F1 strains and their recipient-proficient backcross derivatives. Transconjugant strain nomenclature, and column headings are the same as in Figure S2, with added columns in the F1 analysis for the number of donor segments (donor #), and the percentage of donor DNA transconjugant (% Donor). Backcross (BC) strain names are based on their parent; thus, Km0.1BC is derived from parent Km0.1. Six F1 derivatives were used for the backcross analysis, shown in the far right columns, adjacent to their parental strains. To emphasize the introgression, F1 segments of DNA that were transferred in the backcrosses are alternately colored blue or red. The same colors are used in the final backcross strain to indicate their origin. As above, the length, size, and percent of donor DNA in BC derivatives are indicated. The segment of DNA encoding the *Km* gene is also indicated (green shading), and a column listing the *esx1* genes, if present in the BC strains, has been added.
(XLS)

## Abbreviations

DCT: distributive conjugal transfer
HGT: horizontal gene transfer
*oriT*: origin of transfer
