# Supplementary figures and images for: Distributive Conjugal Transfer in Mycobacteria Generates Progeny with Meiotic-Like Genome-Wide Mosaicism, Allowing Mapping of a Mating Identity Locus
Source: PLoS Biol. 2013 Jul 9;11(7):e1001602. doi: 10.1371/journal.pbio.1001602 (PMC3706393; doi:10.1371/journal.pbio.1001602)

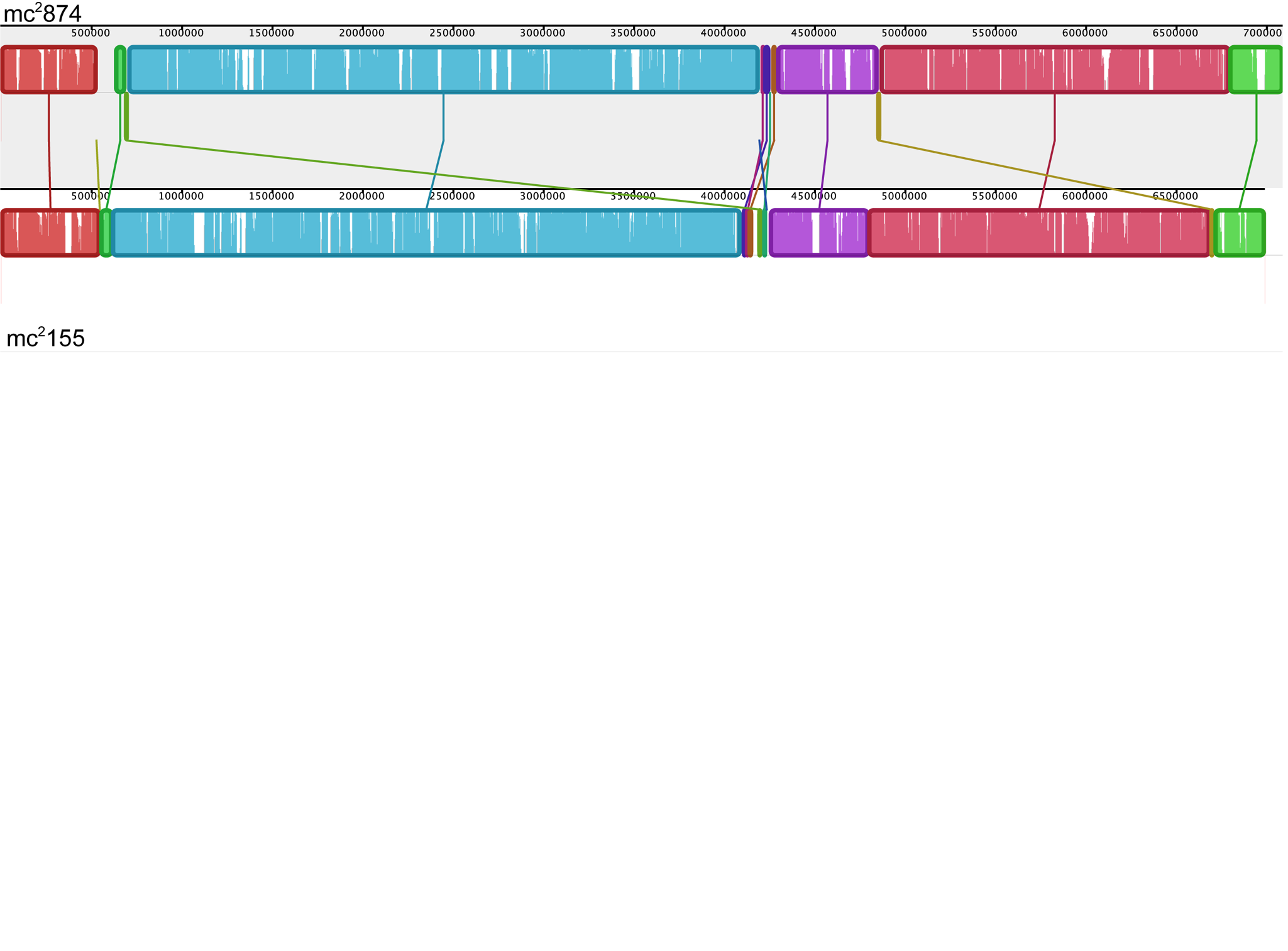

Supplement: Figure S1 — Genome collinearity of the parental strains, mc2155 (donor) and mc2874 (recipient). The circular genomes of the parental strains are depicted in linear form and aligned. Genome sequences for mc2874 were obtained by combining reads from one Illumina and two 454 paired-end libraries (GenBank CM001762). Sequence data are deposited in the EBI/ENA database at http://www.ebi.ac.uk/ena/data/view/ERP002619. A de novo build was assembled into a scaffold, and this nucleotide sequence was aligned (Mauve 2.3.1) with the GenBank/JCVI sequence for mc2155 (CP000480.1) [45],[46]. This figure shows the alignment viewed at Mauve's default, highest stringency setting, thereby displaying even small interruptions. Locally Collinear Blocks (LCBs) are independently colored, with the largest five LCBs comprising nearly all of each genome, and maintaining their order and orientation. The crossed lines between each map indicate modest rearrangements. The sequence data identified 122,186 SNPs (∼1 every 56 bp) between the donor and recipient sequences allowing for easy discrimination between donor and recipient DNA in the transconjugants (see also Figures 1A and S2). (TIF) [file pbio.1001602.s001.tif]

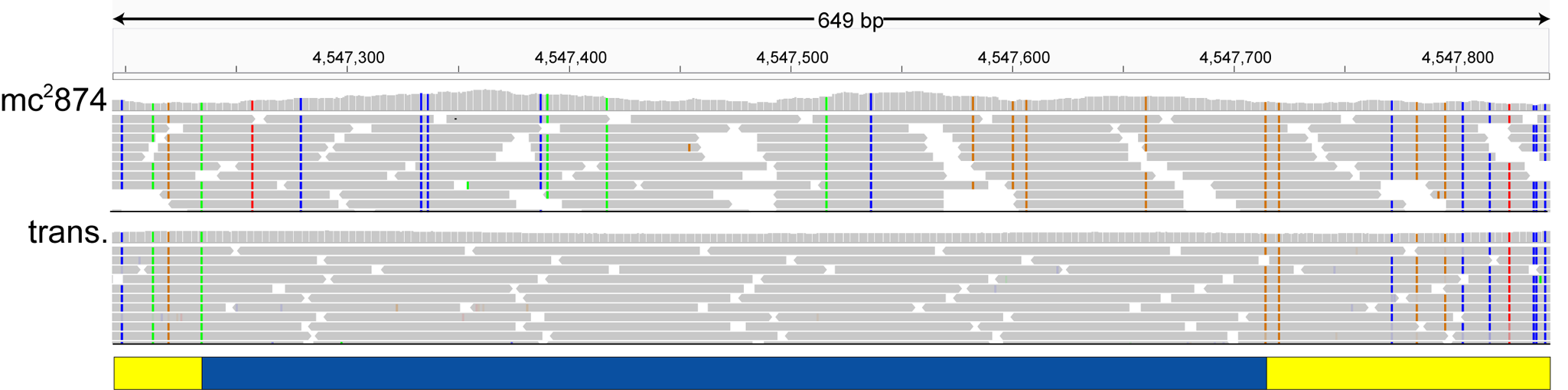

Supplement: Figure S2 — IGV image illustrating donor/recipient junction assignment in a transconjugant. Sequence reads from the recipient strain mc2874 and a transconjugant are shown aligned to the mc2155 reference sequence. A gray bar indicates an individual Illumina sequence read, with the arrow indicating the direction of each read. For simplicity, a depth of 10 reads is shown here, but the average read depth was approximately 50- to 1,000-fold. Gray sequence indicates identity between the sequenced clone and the mc2155 reference genome. Single nucleotide polymorphisms (SNPs) present in sequenced strains appear as colored bases in each read that align vertically with the corresponding polymorphic mc2155 nucleotide. The total SNP content in this 649 bp region is revealed upon mc2874 recipient alignment with the mc2155 reference. Recipient sequence in the transconjugant is conservatively defined by the presence of two consecutive SNPs, and is indicated by the yellow bars below. The left boundary of the replacement donor sequence tract lies between the last recipient SNP present (green) and the next missing SNP (red); as intervening regions match the reference sequence (gray coloration), the donor segment is designated to extend from SNP to SNP, as indicated by the blue bar below. Note that to more clearly discern closely localized donor tracts on the lower resolution Circos plots, successive donor tracts were alternately colored blue or magenta. (TIF) [file pbio.1001602.s002.tif]

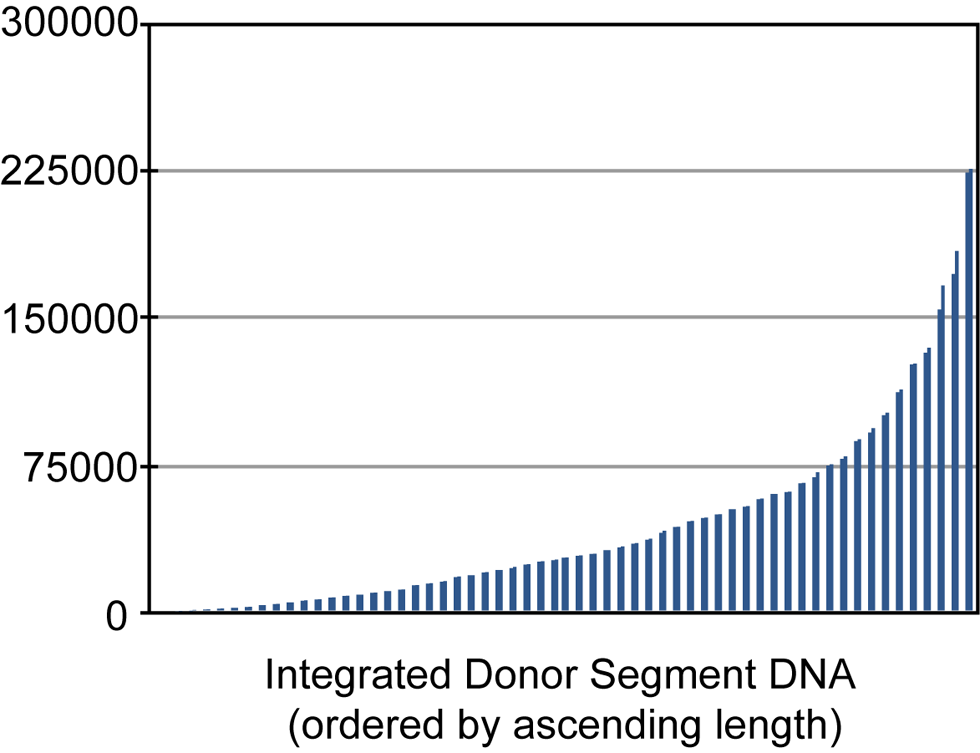

Supplement: Figure S3 — Distribution of donor tract sizes identified in transconjugant genomes. The calculated donor-derived tract sizes for the initial 12 transconjugants are graphically displayed (blue bar represents donor segment length). (TIF) [file pbio.1001602.s003.tif]

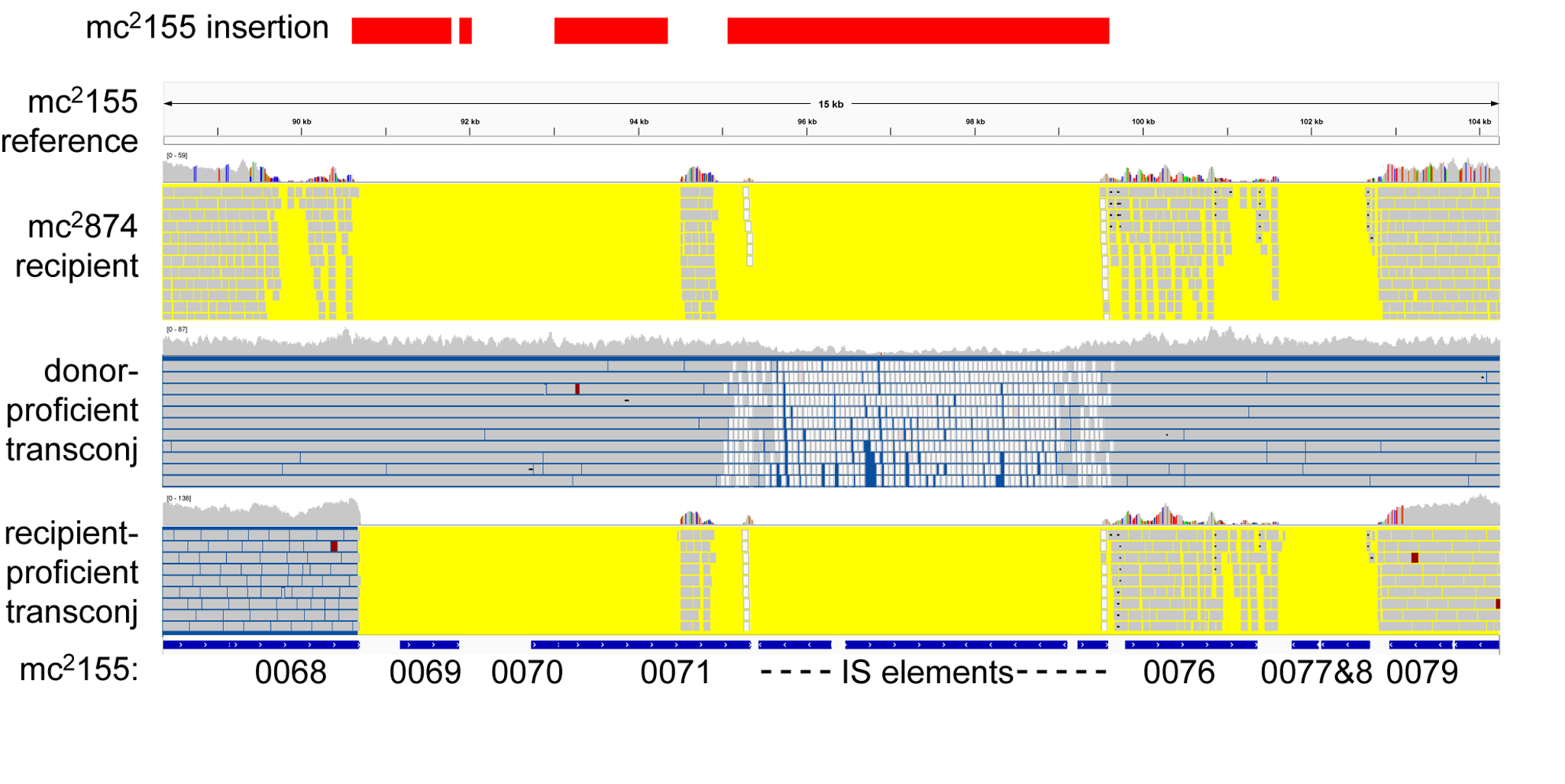

Supplement: Figure S5 — In/dels are transferred in DCT. Whole genome alignment analysis of the mc2155 and mc2874 parental strains by Mauve identified 694 in/dels of >18 bp. The 3′ end of the esx1 locus was identified by Mauve as having insertions in mc2155 (i.e., deleted or divergent in mc2874, indicated by red bars above). Sequence reads aligned to the donor reference viewed in IGV verified that no sequence reads from mc2874 (top alignment, yellow background) mapped to the mc2155 reference sequence in this region, consistent with in/del status. This ∼9 kb region includes donor genes Ms0069 through Ms0071 and a cluster of defective IS elements (Ms0072–0075), displayed at the bottom of the IGV window. Donor sequences from a donor-proficient transconjugant (middle alignment, blue background) have replaced this recipient in/del region, showing that novel sequences can be acquired and incorporated by DCT. Note that reads spanning IS elements in this transconjugant have a lower mapping score (light-shaded reads) because they could map to multiple sites in the genome. Recombination events can occur close to in/del regions, as illustrated by the donor reads in Ms0068 derived from the recipient-proficient transconjugant at the bottom. (TIF) [file pbio.1001602.s005.tif]

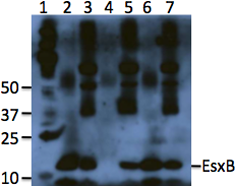

Supplement: Figure S6 — A Western analysis shows that hybrid transconjugants, like their parents, still secrete EsxAB. Culture filtrates and cell pellets were collected as described previously [47]. Following concentration, equivalent cell volumes of each sample were loaded and separated on a 4–12% gradient SDS-PAGE gel. Proteins were transferred to a PVDF membrane and then probed with polyclonal antibodies to detect EsxB. EsxB is secreted by the wild-type strain MKD8 and is therefore detected in both the supernatant (2) and cell pellet (3). In a strain containing a transposon insertion in Ms0062, EsxB is not secreted (4) and is found exclusively in the pellet (5). In transconjugant Km0.1c (Table S2), which contains a mosaic esx1 region, EsxB is found in the supernatant (6) and the pellet (7) as for wild-type. Protein standards are shown in lane 1 and are listed in kilodaltons. (TIF) [file pbio.1001602.s006.tif]
