# Supplementary material for: Distributive Conjugal Transfer in Mycobacteria Generates Progeny with Meiotic-Like Genome-Wide Mosaicism, Allowing Mapping of a Mating Identity Locus
Source: PLoS Biol. 2013 Jul 9;11(7):e1001602. doi: 10.1371/journal.pbio.1001602 (PMC3706393; doi:10.1371/journal.pbio.1001602)
Supplement: Figure S4 — Pairwise alignments of Mid candidate protein sequences between donor and recipient strains of M. smegmatis. A conceptual translation of the six open reading frames comprising the mid candidate regions defined by the combined mapping approaches were globally aligned using a Needleman-Wunsch algorithm (http://www.ebi.ac.uk/Tools/services/web_emboss_needle/). Immediately below each protein identifier (bold text) are input parameters and output statistics. In each alignment, the upper sequence represents the mc2155 (donor) sequence and the bottom is the mc2874 (recipient) sequence. The degree of amino acid conservation is indicated between paired residues: vertical line (identical), dots (similar), or nothing (dissimilar). Horizontal lines represent gaps created by the algorithm to maintain alignment. Similarity statistics for the divergent N-terminus and conserved C-terminus of Msmeg_0071 are listed separately following the full-length alignment of this protein. (DOCX) [file pbio.1001602.s004.docx]

**Msmeg_0069**

########################################

# Program: needle

# Rundate: Fri 9 Dec 2011 13:16:52

# Commandline: needle

# -auto

# -stdout

# -asequence emboss_needle-I20111209-131639-0057-47962883-pg.asequence

# -bsequence emboss_needle-I20111209-131639-0057-47962883-pg.bsequence

# -datafile EBLOSUM62

# -gapopen 10.0

# -gapextend 0.5

# -endopen 10.0

# -endextend 0.5

# -aformat3 pair

# -sprotein1

# -sprotein2

# Align_format: pair

# Report_file: stdout

########################################

#=======================================

#

# Aligned_sequences: 2

# 1: EMBOSS_001

# 2: EMBOSS_001

# Matrix: EBLOSUM62

# Gap_penalty: 10.0

# Extend_penalty: 0.5

#

# Length: 289

# Identity: 82/289 (28.4%)

# Similarity: 113/289 (39.1%)

# Gaps: 69/289 (23.9%)

# Score: 211.5

#

#

#=======================================

EMBOSS_001 1 -MGTPDMRVEEGALGWDPAEVTV--------------PAMPSVGDGPDPL 35

..|.::|| :||||.. |..|...:|.|||

EMBOSS_001 1 MPVTSELRV-------NPAEVRTHGASMEASARAIPEPPDPFKTEGTDPL 43

EMBOSS_001 36 STIIATAVPHVATGVREQVGATRAREERFAENLASARSAYHSTDDGEKQR 85

|..:|.....|.....|.:|.|:|..:..|..:..|...|..:|

EMBOSS_001 44 SKALADKTWQVEAPFIEGIGKTKAEAQETARRIQEAADKYEQSD------ 87

EMBOSS_001 86 IGDAARGIANRANQSSAG----STGIGAFSSAAAVSGARQSDSQFGQLVS 131

|.||... |||.| |||....| .|..|...|..|::.

EMBOSS_001 88 -----RTIAENL-QSSTGRFDASTGTEGTS-----QGGLQGLEQLQQMMQ 126

EMBOSS_001 132 MAMQGGQQALQVPFQLAGMTGQLPQAVQGLVQQTAEWATKGGQAATGPSG 181

|.||..|.|.|||.|:|.|..|:||.|...|||..:.:...||.:.||

EMBOSS_001 127 MPMQAVQMAAQVPTQMAQMAAQVPQTVMQGVQQIGQMSGGTGQDSAGP-- 174

EMBOSS_001 182 SSPRAADLPPDSPTDDEDENLRASPEQDSDEQRKNTGSDASSRGAERAPV 231

::|:. |||.... :...|:| |:|.::.|:.|.....::|||

EMBOSS_001 175 AAPQT-----DSPVGKS----KGGRERD-DQQEQHEGAAADPAFTQQAPV 214

EMBOSS_001 232 ESVRP---QRNPDLKH-----HRNPSTP------PEVAL 256

.|..| ...|:..| ..|.:.| |.:.|

EMBOSS_001 215 TSQVPGAAASEPEAGHSEGRPRANGTVPRPVPSDPAIVL 253

**Msmeg_0070**

########################################

# Program: needle

# Rundate: Fri 9 Dec 2011 13:19:42

# Commandline: needle

# -auto

# -stdout

# -asequence emboss_needle-I20111209-131941-0822-27912293-pg.asequence

# -bsequence emboss_needle-I20111209-131941-0822-27912293-pg.bsequence

# -datafile EBLOSUM62

# -gapopen 10.0

# -gapextend 0.5

# -endopen 10.0

# -endextend 0.5

# -aformat3 pair

# -sprotein1

# -sprotein2

# Align_format: pair

# Report_file: stdout

########################################

#=======================================

#

# Aligned_sequences: 2

# 1: EMBOSS_001

# 2: EMBOSS_001

# Matrix: EBLOSUM62

# Gap_penalty: 10.0

# Extend_penalty: 0.5

#

# Length: 403

# Identity: 139/403 (34.5%)

# Similarity: 195/403 (48.4%)

# Gaps: 40/403 ( 9.9%)

# Score: 591.5

#

#

#=======================================

EMBOSS_001 1 --------------MLAGLRRRGSMTT----PPGPEIPPPHQGGFYSAGH 32

.|.|....||..: |.||..||| ||.

EMBOSS_001 1 MTLPPPPGSFGQQPPLGGHSGGGSPESWPQYPGGPTSPPP-------AGP 43

EMBOSS_001 33 HPQRP---WPETPPPKTRGG-VKWMLGAVALLAVVGVTVAVTLAVTGKDK 78

.|..| |...|.|...|| |:||||.:|::..:.:.|.||:.|...|.

EMBOSS_001 44 PPWGPQPQWANGPTPPNGGGKVRWMLGGLAVVLAIALAVVVTVLVVRPDA 93

EMBOSS_001 79 RDAIPPGS--GVSGSPTASDIASADDSGPVSVITEDPTCAAQGPILETFA 126

.: || ..:|.| ||..||.:|.||||:||:||||.....|...:.

EMBOSS_001 94 EN----GSKDEKTGGP-ASGFASENDDGPVSIITDDPTCDGWARITREYN 138

EMBOSS_001 127 AQQSQL-WVERDPALGRESWSPELRADYEKVGKAMRTAADQVAQLAKITP 175

|:.:.: |.|||.::...:|:||.|..|..:.|||.||||....|.|.||

EMBOSS_001 139 AESTAVRWAERDASIPANAWTPEQRDMYNTMAKAMTTAADHTEALIKQTP 188

EMBOSS_001 176 HRAMRELYEQFIAYARAYADNIPNYTPPTDNLARVAVTAADAISYICAAV 225

||||||||:||.||...:...||.|.|..:....|....|::.:.||.|:

EMBOSS_001 189 HRAMRELYQQFSAYVHVFVPLIPAYVPENNRFVPVINALANSATDICTAI 238

EMBOSS_001 226 SYGSAAARAPLVENRPAPTNVAPLGNPSEPERFLTAPNPVCGEWSSVLNA 275

.:.||:..|..:.:...|:.:|........:.||...||||.:.:|.:.|

EMBOSS_001 239 EFRSASTFAGRIPSVDPPSRLASTSGADASDLFLGDGNPVCADLASAVVA 288

EMBOSS_001 276 FQTDTTEWLKTDPDISSSQWSIEQKQINENVIPIMKRFANQLYLLGKDSw 325

|...|..|...||.:.:::||.:.:.:.::|.|:|...|:.|..||:.|

EMBOSS_001 289 FDEQTRAWQALDPKLPAAEWSPDHRAVMDDVAPVMSANADNLERLGRAS- 337

EMBOSS_001 326 GNPTFRDIADLSVQYRLAYVAAIPTYTPADKYLANASIRLATMANVACRA 375

.|....|...|:.||:..||.|||||:.||..|......|....|..|:|

EMBOSS_001 338 DNAIVEDFTVLAAQYQRGYVEAIPTYSSADNVLWQVVASLVKAVNSGCKA 387

EMBOSS_001 376 AAD 378

:

EMBOSS_001 388 S-- 388

**Msmeg_0071**

########################################

# Program: needle

# Rundate: Fri 9 Dec 2011 13:21:16

# Commandline: needle

# -auto

# -stdout

# -asequence emboss_needle-I20111209-132115-0176-1112722-pg.asequence

# -bsequence emboss_needle-I20111209-132115-0176-1112722-pg.bsequence

# -datafile EBLOSUM62

# -gapopen 10.0

# -gapextend 0.5

# -endopen 10.0

# -endextend 0.5

# -aformat3 pair

# -sprotein1

# -sprotein2

# Align_format: pair

# Report_file: stdout

########################################

#=======================================

#

# Aligned_sequences: 2

# 1: EMBOSS_001

# 2: EMBOSS_001

# Matrix: EBLOSUM62

# Gap_penalty: 10.0

# Extend_penalty: 0.5

#

# Length: 834

# Identity: 409/834 (49.0%)

# Similarity: 479/834 (57.4%)

# Gaps: 137/834 (16.4%)

# Score: 1719.5

#

#

#=======================================

EMBOSS_001 1 ----------------------------------MVTIQPDASEYGG--- 13

|:.:.......||

EMBOSS_001 1 MCRSDSNLLLCGQCPLAGCCLTRQSRQQRVQGIVMMGLGKLEKPKGGHFD 50

EMBOSS_001 14 QTVTGAAWPNIDESVLASAASDLEAVANHLRDNVVPSAGRQKMKLA-DSW 62

:.|...|||..:||..::..:::..: ||::..| :||

EMBOSS_001 51 RMVGPGAWPETEESEFSNRQAEILGI-------------RQRLTGAKESW 87

EMBOSS_001 63 ---------EGKGADSALDEANAIIGEHEQNEVEAREAAAKLRR-MEFAV 102

|...|.::...|||.:.:|.::.|.......|:.: ...|:

EMBOSS_001 88 QTHQSYLDSEATWAGTSARAANARVEQHTKSMVAHEHQLEKVEKWCGEAI 137

EMBOSS_001 103 AVAKTLANQTAEQVQNDCQRIMDEGQPSEGEDTRFERVATRIRE-GYDDN 151

.:..|:.......|.:..:.|.:....::..:|.......:|:: .||.|

EMBOSS_001 138 GLIGTVKEAIIGTVNSAVEAINEAAADADKNETDASSKIEKIKDLAYDFN 187

EMBOSS_001 152 VRMVAEVSHQLAADL----GIVPPEPWGHPGSPAR-----TPQTPDQH-- 190

..:| |..||. ..:|.|| |..|.. .|.|..|.

EMBOSS_001 188 KSIV-----QWGADTVQGKEDIPSEP---PKLPKDIFKNVEPATGSQDGE 229

EMBOSS_001 191 -PDGQLGTVDSGSSAPAPPPSLFAPPPPSRNADRPSQ----LGTEVAAKA 235

.|||...:...||.......:.|...|||..|...| || |.|:..

EMBOSS_001 230 IRDGQADVLFMNSSLGGDASDVRASVKPSRPVDTWEQDNVGLG-EDASGV 278

EMBOSS_001 236 EPTPSLE-TVQSGSPVPPDPLVGGEAP-ARSNPPFAQ------PHSDAPS 277

.|||::| .|:...|| :..:|||.| .|..||..| |.:.||:

EMBOSS_001 279 RPTPAIEPPVEQVEPV--NSGLGGEQPDIRPAPPIPQPVLGRPPSAPAPT 326

EMBOSS_001 278 TSGASTP-PAVGP-APAPT------TGSGTMRPQAPAQALPPG-HLDSLS 318

..|.|.| .|:.| ||.|. ||.|.....||:..|..| .|....

EMBOSS_001 327 AGGPSGPGSAISPSAPTPVSSSGPPTGGGGGTSIAPSSPLSSGSSLGGSG 376

EMBOSS_001 319 PATSNEISSNAVSPSAFGAKSGTPLEQFQKGLAD-------AAKTGGSPQ 361

...:.:.|:.|:..:..|.....||:||.:..:| ||.|||

EMBOSS_001 377 AEQAADASAAALGQNPVGRAPVDPLQQFTQSFSDSAGTPVHAASTGG--- 423

EMBOSS_001 362 TLSTAPSQPLGAPPTTQPLGAAPPTAGPAAPPTT--GGPPAPV-AQAAGG 408

.:.|..|| .||.|.|.|.:...|...:.||: ..|.||| ..||||

EMBOSS_001 424 --GSVPPPPL-TPPPTVPAGESMGPASAHSTPTSLNSAPAAPVQPPAAGG 470

EMBOSS_001 409 PGGGAGPAPVAPPLSGGVPGGAVPLGPPPTPPPAAPVTTPPLASGAPVAP 458

|.|.|...||||| ||||||||||||||..||.|:..|.|.

EMBOSS_001 471 PMGAASGMPVAPP----------PLGPPPTPPPAAPVAAPPPAAAPPPAQ 510

EMBOSS_001 459 TGAAAGAAGGGGAQVAPIPVSAARAERDLAQRAVRRSGVDPMETARRIAA 508

..|| |||||||||||||||||||||||||||||||||||||||||

EMBOSS_001 511 PNAA-----GGGAQVAPIPVSAARAERDLAQRAVRRSGVDPMETARRIAA 555

EMBOSS_001 509 ALNAPGMTNVEDFKFFWVTGLTADGKIVVANNYGIAYIPQQVHLPEQVYM 558

||||||||||||||||||||||||||||||||||||||||||||||||:|

EMBOSS_001 556 ALNAPGMTNVEDFKFFWVTGLTADGKIVVANNYGIAYIPQQVHLPEQVHM 605

EMBOSS_001 559 ASADESISPAERASWVNEPIVAVQRWAEHNGRVLRAVIATEDQLKNSDAG 608

|||||||||||||.|||||||||||||||:|:.|||||||||||||||||

EMBOSS_001 606 ASADESISPAERARWVNEPIVAVQRWAEHHGKNLRAVIATEDQLKNSDAG 655

EMBOSS_001 609 VHHEVLRPEDIPENGKMAGRDRLQVIAPDVSSQLAKIGDADLVSVLPPAP 658

|||||||||||||.|||||||||||||||||:|||::.|.|||.:|||||

EMBOSS_001 656 VHHEVLRPEDIPEGGKMAGRDRLQVIAPDVSAQLARVSDTDLVKILPPAP 705

EMBOSS_001 659 ADSNPPEDRRKSLWDNVWKPLASRSAKRGERHLTAFVAYAAHAQEHALYA 708

||:||||||||.|||||||||||||.|||||||.||||||||||||||||

EMBOSS_001 706 ADANPPEDRRKLLWDNVWKPLASRSTKRGERHLAAFVAYAAHAQEHALYA 755

EMBOSS_001 709 AHTAALPDEQRQAIREFIYWQHVGQLTADALAPA 742

||||||||:||||||||||||||||||||||:||

EMBOSS_001 756 AHTAALPDDQRQAIREFIYWQHVGQLTADALSPA 789

#---------------------------------------

#---------------------------------------

**N-terminus only:**

########################################

# Program: needle

# Rundate: Fri 9 Dec 2011 13:27:26

# Commandline: needle

# -auto

# -stdout

# -asequence emboss_needle-I20111209-132725-0283-66418926-pg.asequence

# -bsequence emboss_needle-I20111209-132725-0283-66418926-pg.bsequence

# -datafile EBLOSUM62

# -gapopen 10.0

# -gapextend 0.5

# -endopen 10.0

# -endextend 0.5

# -aformat3 pair

# -sprotein1

# -sprotein2

# Align_format: pair

# Report_file: stdout

########################################

#=======================================

#

# Aligned_sequences: 2

# 1: EMBOSS_001

# 2: EMBOSS_001

# Matrix: EBLOSUM62

# Gap_penalty: 10.0

# Extend_penalty: 0.5

#

# Length: 559

# Identity: 153/559 (27.4%)

# Similarity: 213/559 (38.1%)

# Gaps: 137/559 (24.5%)

# Score: 389.5

#

#

#=======================================

**C-terminus only:**

########################################

# Program: needle

# Rundate: Fri 9 Dec 2011 13:30:43

# Commandline: needle

# -auto

# -stdout

# -asequence emboss_needle-I20111209-133029-0951-30296974-pg.asequence

# -bsequence emboss_needle-I20111209-133029-0951-30296974-pg.bsequence

# -datafile EBLOSUM62

# -gapopen 10.0

# -gapextend 0.5

# -endopen 10.0

# -endextend 0.5

# -aformat3 pair

# -sprotein1

# -sprotein2

# Align_format: pair

# Report_file: stdout

########################################

#=======================================

#

# Aligned_sequences: 2

# 1: EMBOSS_001

# 2: EMBOSS_001

# Matrix: EBLOSUM62

# Gap_penalty: 10.0

# Extend_penalty: 0.5

#

# Length: 275

# Identity: 256/275 (93.1%)

# Similarity: 266/275 (96.7%)

# Gaps: 0/275 ( 0.0%)

# Score: 1342.0

#

#

#=======================================

**Msmeg_0076**

########################################

# Program: needle

# Rundate: Mon 23 Apr 2012 20:36:03

# Commandline: needle

# -auto

# -stdout

# -asequence emboss_needle-I20120423-203600-0329-51308596-oy.asequence

# -bsequence emboss_needle-I20120423-203600-0329-51308596-oy.bsequence

# -datafile EBLOSUM62

# -gapopen 10.0

# -gapextend 0.5

# -endopen 10.0

# -endextend 0.5

# -aformat3 pair

# -sprotein1

# -sprotein2

# Align_format: pair

# Report_file: stdout

########################################

#=======================================

#

# Aligned_sequences: 2

# 1: EMBOSS_001

# 2: EMBOSS_001

# Matrix: EBLOSUM62

# Gap_penalty: 10.0

# Extend_penalty: 0.5

#

# Length: 526

# Identity: 486/526 (92.4%)

# Similarity: 502/526 (95.4%)

# Gaps: 6/526 ( 1.1%)

# Score: 2561.0

#

#

#=======================================

EMBOSS_001 1 MSEELQYELPGLERKAHECESTRPEGPGDATKPDELATTASVYSKLMASA 50

|||||:|||||||||||||||||||||||||||||||||||||.||||||

EMBOSS_001 1 MSEELKYELPGLERKAHECESTRPEGPGDATKPDELATTASVYGKLMASA 50

EMBOSS_001 51 AKLKATFAAGDREGERIAAAIRAAAGAYQKIEEQKAAELSRQMNGSDAPP 100

||||||||||||||:||||||||||||||||||||||||:||||||||||

EMBOSS_001 51 AKLKATFAAGDREGKRIAAAIRAAAGAYQKIEEQKAAELNRQMNGSDAPP 100

EMBOSS_001 101 PAAEAVVPDMSGIPGPLAIPSMEYPSAAAAADEMDWEAAARIIHSGDTQA 150

|||||||||||||||||.||||||||||||||||||||||||||||||||

EMBOSS_001 101 PAAEAVVPDMSGIPGPLTIPSMEYPSAAAAADEMDWEAAARIIHSGDTQA 150

EMBOSS_001 151 LSMKYFRDQWRDYQSTLEGHGRHFANPAEGWAGAAAETCAEAQRRLSTWW 200

|||||||||||||||||||||||||||||||:||||||||||||||||||

EMBOSS_001 151 LSMKYFRDQWRDYQSTLEGHGRHFANPAEGWSGAAAETCAEAQRRLSTWW 200

EMBOSS_001 201 ADMGAECGRLAQEATTFVDAHDKLVANHPTLENVREFEETEWASEWDRQN 250

||||||||||||||||||||||||||||||:::|:.|||.||.||||||.

EMBOSS_001 201 ADMGAECGRLAQEATTFVDAHDKLVANHPTMDDVKAFEEAEWESEWDRQY 250

EMBOSS_001 251 AWAMLQEQSEDALEAYANGSQIQEIRPGKPPSIGGLPAVNDGDVQASPTS 300

|||:.||:||||||||||||||||||||||||||||||||||||||:|||

EMBOSS_001 251 AWAIKQEKSEDALEAYANGSQIQEIRPGKPPSIGGLPAVNDGDVQATPTS 300

EMBOSS_001 301 APGGPGGPGSGTPG--GGGA--GGGGGTPEMPELPSTDPSMSPMSANSAG 346

..|||||||||:.| |||| |.|||||||||||||||||||||.||||

EMBOSS_001 301 TTGGPGGPGSGSGGGSGGGASGGSGGGTPEMPELPSTDPSMSPMSTNSAG 350

EMBOSS_001 347 EEQSSGSPSSGGSPS-GSPSGGSPSGGGAPSGAGMPDGGLPSDLPGGPDI 395

|||||||||||||.| |||||||||||||||..|||:||||:|:||||||

EMBOSS_001 351 EEQSSGSPSSGGSSSGGSPSGGSPSGGGAPSSGGMPEGGLPTDMPGGPDI 400

EMBOSS_001 396 PGLDDPSLKPASAGGGGGGGV-GGGGGGMPAAPLGPAVGADSVSPSPSST 444

||||||||||||||||||||| ||||||||||||||||||||||||||||

EMBOSS_001 401 PGLDDPSLKPASAGGGGGGGVGGGGGGGMPAAPLGPAVGADSVSPSPSST 450

EMBOSS_001 445 RGGGVGVPGGPGGGAGGMMGGGMGGMGAGHGQGQGKEKKRDPKLAPDEDL 494

||||||||.|.|||||||||||||||||||||||||||||||||||||||

EMBOSS_001 451 RGGGVGVPTGTGGGAGGMMGGGMGGMGAGHGQGQGKEKKRDPKLAPDEDL 500

EMBOSS_001 495 YTEDRAHTEGVIGHRARREKDSGKQQ 520

|||||||:|||||||.||||||||||

EMBOSS_001 501 YTEDRAHSEGVIGHRPRREKDSGKQQ 526

#---------------------------------------

#---------------------------------------

**Msmeg_0077**

#=======================================

#

# Aligned_sequences: 2

# 1: EMBOSS_001

# 2: EMBOSS_001

# Matrix: EBLOSUM62

# Gap_penalty: 10.0

# Extend_penalty: 0.5

#

# Length: 105

# Identity: 74/105 (70.5%)

# Similarity: 85/105 (81.0%)

# Gaps: 6/105 ( 5.7%)

# Score: 373.0

#

#

#=======================================

EMBOSS_001 1 MSGDLKVTAAELRELSERQRQIVENIATAAQATNNTTALVTVTHGPVCAP 50

::|::|||:||||||:..::|.|||..|.||||||.|||.||||

EMBOSS_001 1 ------mSASDLREVSERQRQVASSVAAAAQTVNGTTALVTATHGLVCAP 44

EMBOSS_001 51 TIAAIGAAGFSRDAAAAAMQGVSTSLAEKLDEAATNYERTDAAKAGDLDG 100

|||||||||||||||||||:.||:||||||||||.||||||..:|.||||

EMBOSS_001 45 TIAAIGAAGFSRDAAAAAMRSVSSSLAEKLDEAAANYERTDIDEAADLDG 94

EMBOSS_001 101 EMHGS 105

||||.

EMBOSS_001 95 EMHGP 99

**Msmeg_0078**

# Aligned_sequences: 2

# 1: EMBOSS_001

# 2: EMBOSS_001

# Matrix: EBLOSUM62

# Gap_penalty: 10.0

# Extend_penalty: 0.5

#

# Length: 244

# Identity: 159/244 (65.2%)

# Similarity: 185/244 (75.8%)

# Gaps: 23/244 ( 9.4%)

# Score: 824.0

#

#

#=======================================

EMBOSS_001 1 MSDFGDLYDIANNWYSGYSHTVGMAEDTRYTAAGLGANAISFGQSLARDG 50

|.||......|||||||.|..|.|::|

EMBOSS_001 1 -----------------------MKEDPGSAFFGLGANAIDFAHSAAQEG 27

EMBOSS_001 51 AQKLGDTKLAAAAATPIIAFGLRTMTIMSNLTGVEGPEHGDRYGQGAEAF 100

|:|.||.:.|||.|||||:.|:|.||:|||:||.||||.|||||:||:||

EMBOSS_001 28 AEKFGDLRSAAALATPIISMGIRIMTVMSNMTGFEGPERGDRYGRGADAF 77

EMBOSS_001 101 SGVSSGLDGTRSPDSWEGSSSDAYSDRNREQKERAALMAETDRVVKEVLD 150

:.||..||||||||||:||||:|||||||||:|||.|||:||..:|||||

EMBOSS_001 78 ASVSDDLDGTRSPDSWDGSSSEAYSDRNREQQERAQLMAKTDAEIKEVLD 127

EMBOSS_001 151 KEAGEIEDTRRQIDHQMTELTWLIPAAIAAKFWNAPPGSGEIASQIIQWG 200

:||.:|:|||.||..|::|||:|||.|:|||.||.||||||||||.|||.

EMBOSS_001 128 EEALQIKDTRNQISRQISELTYLIPVALAAKLWNVPPGSGEIASQAIQWA 177

EMBOSS_001 201 GVAKTLPIATQRMYRMIADSSENATLIRRAGATYDRIAAEAQAQ 244

|.|||:||||||||||::|||.||||||||||||||||||||||

EMBOSS_001 178 GFAKTVPIATQRMYRMVSDSSHNATLIRRAGATYDRIAAEAQAQ 221

**Figure S3. Pairwise alignments of sex-determining candidate protein sequences between the donor and recipient strains of *M. Smegmatis*.** A conceptual translation of the six open reading frames comprising the *sxd* candidate regions defined by the combined GWAS and GWIS mapping data were globally aligned using a Needleman-Wunsch algorithm (<http://www.ebi.ac.uk/Tools/services/web_emboss_needle/>). Immediately below each protein identifier (**bold text**) are input parameters and output statistics. In each alignment, the upper sequence represents the mc^2^155 (donor) sequence and the bottom is the mc^2^874 (recipient) sequence. The degree of amino acid conservation is indicated between paired residues: vertical line (identical), dots (similar), or nothing (dissimilar). Gaps created by the algorithm to maintain alignment are indicated by horizontal lines.
