# Supplementary material for: Distributive Conjugal Transfer in Mycobacteria Generates Progeny with Meiotic-Like Genome-Wide Mosaicism, Allowing Mapping of a Mating Identity Locus
Source: PLoS Biol. 2013 Jul 9;11(7):e1001602. doi: 10.1371/journal.pbio.1001602 (PMC3706393; doi:10.1371/journal.pbio.1001602)
Supplement: Table S1 — Primers used to verify transferred donor SNPs in transconjugant genomes. The primers, their genome coordinates, used for each transconjugant are listed. Sanger sequencing of the PCR product verified the presence of uniquely transferred donor SNPs in transconjugant genomes. Multiple informative SNPs present in each amplicon to facilitated unambiguous parental origin identification. Two PCR clones were sequenced from each transconjugant strain to avoid potential complications from PCR errors. (DOCX) [file pbio.1001602.s007.docx]

| Transconjugant |  | Primer Sequence | Position |
| --- | --- | --- | --- |
| Km2.2b | FWD | CACCGCGGACTGGATCG | 1868734 |
|  | REV | TGCTGGCACTGGGGCTG | 1869284 |
| Km0.1a | FWD | ACTCGTCGAGATCCAGCC | 5314352 |
|  | REV | CGATCTGCGGGTCTATCC | 5314922 |
| Km6.4b | FWD | CGTTGCGCGACCGCTTC | 4485118 |
|  | REV | CTGGAACGCCGGATCGG | 4485610 |
| Km1.4 | FWD | GGTCGACGCTGACCGAG | 1528222 |
|  | REV | GTGTCGGCGCGCAGATG | 1528681 |
| Km6.9b | FWD | CTGCCGACGACGACGTC | 4888016 |
|  | REV | GAACCTGGCACCCGGTG | 4888539 |
| Km6.4a | FWD | GCATGTCGGCGACCTCG | 6836263 |
|  | REV | CTGCACTTCGGCCTGGC | 6836644 |
| Km6.9c | FWD | GGCCATCCGCAGTTCCC | 6673722 |
|  | REV | GTTCTACCGGCACCGGC | 6674231 |
| Km0.1b | FWD | CGACGGTCACCGGTACG | 3726503 |
|  | REV | CACGGTCACACCCGACG | 3726960 |
| Km5.7 | FWD | GCGGTGCCCAGTGTCTC | 2639940 |
|  | REV | CGTCCGAGAACGGCCAC | 2640489 |
| Km0.8 | FWD | GTGTGTGCACGGTGCCG | 2353727 |
|  | REV | GTGGCACGCGGGCTTG | 2354238 |
| Km3.2 | FWD | CGGGAGGTCAAGGACG | 3106841 |
|  | REV | TTCGTGGATCTGCAGCCC | 3107359 |
| Km3.8 | FWD | GCTGATGCATGCGCGTG | 3685500 |
|  | REV | CTCGCAGGTCCTCATCGT | 3686022 |
| Km4.5b | FWD | CACGGCAGGCCGCTGG | 3547411 |
|  | REV | GCGATCGAACCGGGCAC | 3547934 |
| Km2.2a | FWD | CGACGGTGAACTCGTCG | 2318883 |
|  | REV | CGTGTTCGGGTCCGGTT | 2319408 |
