# Supplementary material for: Distributive Conjugal Transfer in Mycobacteria Generates Progeny with Meiotic-Like Genome-Wide Mosaicism, Allowing Mapping of a Mating Identity Locus
Source: PLoS Biol. 2013 Jul 9;11(7):e1001602. doi: 10.1371/journal.pbio.1001602 (PMC3706393; doi:10.1371/journal.pbio.1001602)
Supplement: Table S3 — Transfer frequencies of F1 transconjugants. Transfer frequencies are the number of transconjugants divided by the number of donor cells. These frequencies are the average of at least two independent matings, which were carried out in parallel with a positive control (MKD6 and MKD8, [8]). The threshold of detection is ∼1 transfer event per 108 donor or recipient parental cells. (DOCX) [file pbio.1001602.s009.docx]

| Transconjugant | Donor efficiency | Recipient efficiency |
| --- | --- | --- |
| Km6.4b | 4x10^-5^ | <10^-8^ |
| Km0.1a | 5x10^-5^ | <10^-8^ |
| Km0.1b | 3x10^-5^ | <10^-8^ |
| Km2.2b | 2x10^-6^ | <10^-8^ |
| Km1.4 | 1x10^-5^ | <10^-8^ |
| Km5.7 | 7x10^-5^ | <10^-8^ |
| Km0.8 | 2x10^-5^ | <10^-8^ |
| Km6.9a | 5x10^-5^ | <10^-8^ |
| Km4.5a | 5x10^-4^ | <10^-8^ |
| Km3.8 | <10^-8^ | 3x10^-6^ |
| Km6.4a | <10^-8^ | 1x10^-6^ |
| Km2.2a | <10^-8^ | 5x10^-6^ |
| Km3.2 | <10^-8^ | 2x10^-5^ |
| Km6.9c | <10^-8^ | 1x10^-5^ |
| Km6.9e | <10^-8^ | 2x10^-5^ |
| Km4.5b | <10^-8^ | 1x10^-6^ |
